# Self-specific processing in the meditating brain: a MEG neurophenomenology study

**DOI:** 10.1093/nc/niw019

**Published:** 2016-10-10

**Authors:** Yair Dor-Ziderman, Yochai Ataria, Stephen Fulder, Abraham Goldstein, Aviva Berkovich-Ohana

**Affiliations:** 1 Gonda Brain Research Center, Bar-Ilan University, Ramat-Gan, Israel;; 2 Neurobiology Department, Weizmann Institute of Science, Rehovot, Israel;; 3 Founder, Senior Teacher, Israel Insight Society (Tovana), Israel;; 4 Psychology Department, Bar-Ilan University, Ramat-Gan, Israel;; 5 Faculty of Education, The Safra Brain Research Center for the Study of Learning Disabilities, University of Haifa, Haifa, Israel; 6 Faculty of Humanities and Social Sciences, Tel-Hai Academic College, Upper Galilee, Israel

**Keywords:** **:** self-specific processes, minimal self, MEG, neurophenomenology, meditation, beta band, parietal cortex

## Abstract

Self-specific processes (SSPs) specify the self as an embodied subject and agent, implementing a functional self/nonself distinction in perception, cognition, and action. Despite recent interest, it is still undetermined whether SSPs are all-or-nothing or graded phenomena; whether they can be identified in neuroimaging data; and whether they can be altered through attentional training. These issues are approached through a neurophenomenological exploration of the sense-of-boundaries (SB), the fundamental experience of being an ‘I’ (self) separated from the ‘world' (nonself). The SB experience was explored in collaboration with a uniquely qualified meditation practitioner, who volitionally produced, while being scanned by magnetoencephalogram (MEG), three mental states characterized by a graded SB experience. The results were then partly validated in an independent group of 10 long-term meditators. Implicated neural mechanisms include right-lateralized beta oscillations in the temporo-parietal junction, a region known to mediate the experiential unity of self and body; and in the medial parietal cortex, a central node of the self's representational system. The graded nature as well as the trainable flexibility and neural plasticity of SSPs may hold clinical implications for populations with a disturbed SB.

## Introduction

In the last decade, cognitive neuroscience has widened its exploration of the neural processes giving rise to self-experience from processes that evaluate certain features in relation to one’s perceptual image or mental concept of oneself (self-related processes, SRP), to processes that specify the self as an embodied subjective knower and agent (self-specific processes, SSPs) (Christoff *et al.*, 2011; Blanke, 2012; Seth, 2013). SRP processes, also known as “extended” (Damasio, 1999) or “narrative” (Gallagher, 2000) –self processes, have so far received the bulk of the neuroimaging community’s attention and have been shown to be closely linked to the subjective content and neural activity attributed to the default-mode network (DMN, Raichle *et al.*, 2001), in particular involving medial regions (Gusnard *et al.*, 2001; Northoff *et al.*, 2006; Buckner *et al.*, 2008; Andrews-Hanna *et al.*, 2010). These reference the “self-as-object” (James, 1890) and typically involve tasks assessing one’s personality, traits, name, or appearance. As such, they include higher-order cognitive functions such as evaluation, judgment, and reflective thought (Legrand and Ruby, 2009; Christoff *et al.*, 2011; Northoff *et al.*, 2011). SSPs reference the “self-as-subject” (James, 1890). They implement a functional self/nonself distinction in perception, action, cognition, and emotion (Christoff *et al.*, 2011). In line with this, self-specific features have been defined as being exclusive and non-contingent, meaning that they characterize oneself and no-one else, and that “changing” or “losing” them entail “changing” or “losing” the distinction between self and nonself. So far, neurocognitive attempts to investigate SSPs have employed paradigms which “changed” self-specific features, resulting in an altered sense of agency and body-ownership (Farrer and Frith, 2002; Tsakiris *et al.*, 2008, 2010; Nahab *et al.*, 2011; Chambon *et al.*, 2014), or self-identification, location, and perspective (Arzy *et al.*, 2006; Blanke and Metzinger, 2009; Blanke, 2012; Guterstam *et al.*, 2015). These studies highlight the involvement of the temporo-parietal junction. However, no neuroimaging study to date has reported on the volitional reduction of self-specific features, or on the neural substrate underlying conscious experience devoid of the felt distinction between the “self” and “world.”

Other important but still unresolved questions include: are SSPs all-or-nothing or graded phenomena, and can attentional training drive SSP-neuroplasticity? Christoff *et al.* (2011) argue that addressing these questions necessitate broadening our understanding of the self-experience by incorporating subjective measures into neuroimaging protocols, as previously emphasized by Varela’s neurophenomenology research program (Varela *et al.*, 1991; Varela, 1996). In particular, Varela suggested collaborating with highly skilled meditation practitioners as “… mindful awareness practices can provide a natural bridge between cognitive science and human experience (phenomenology). Particularly impressive to us is the convergence that we have discovered among the main themes concerning the self and the relation between subject and object.” (Varela *et al.*, p. 33).

It should be noted that contemplation-induced loss of self is different from loss of self as evidenced in psychopathology. The former is basic to the sense of felt meaning and purpose in human existence, while the latter reflects the extremity of its collapse (Hunt, 2007). Buddhist notions of selflessness emphasize flexibility in the perception of the self, which leads to eudemonic happiness and optimal functioning (Dambrun and Ricard, 2011). The meditation styles directly aimed at achieving such states are categorized under the “Deconstructive family” (Dahl *et al.*, 2015). These target the implicit belief that the self is static, enduring and unitary, and replacing identification with it by identification with the phenomenon of experiencing itself (Dalai Lama, 1997).

The current article addresses these issues through a neurophenomenological exploration (Lutz *et al.*, 2002; Thompson *et al.*, 2005) of the sense-of-boundaries (SB), the fundamental division of the field of experience to a “self” versus a “world.” By collaborating with a uniquely qualified meditation practitioner (see section “Methods”), the SB experience was volitionally and repeatedly produced as a graded phenomenon, from a normal SB (SB1) to a state where the SB began to dissolve (SB2) and finally to a state where the SB disappeared (SB3), while brain activity was recorded using magnetoencephalogram (MEG). These states were investigated using a first-person approach where in-depth phenomenological interviews were conducted, and the collected data were analyzed using the grounded approach (explained in great detail in Ataria *et al.*, 2015). The phenomenological interview method elicits from interviewees their own descriptions in their own words, “bracketing out” predetermined descriptive categories and concepts (such as Buddhist jargon). In addition, the grounded theory approach considers data with no hypotheses or categories fixed at the outset, staying as close as possible to the data. Through this process, nine categories of experience that diminished during the shifts between the three SB stages were identified. These are presented and discussed in detail in Ataria *et al.* (2015). The categories are summarized in Table 1 below. The main conclusions of the phenomenological inquiry were that the SB should be defined in terms of flexibility, rather than location; and that the more flexible the SB, the weaker these phenomenal categories become, some dissolving completely and some maintaining a very weak presence.

**Table 1:** The nine phenomenal categories and their expression during the SB stages

Category	Brief explanation	Degree during different stages		
		SB1	SB2	SB3
Internal vs. external	As the SB becomes more flexible, it is much less clear what is “inside” and what is “outside”; the experience of in versus out fades away. From the perspective of the SB, the concept of in versus out can be defined in terms of “priority.” There is no strict line between inside and outside, instead there is a continuum: something more important is “closer” and as things become less important, they grow increasingly distant.	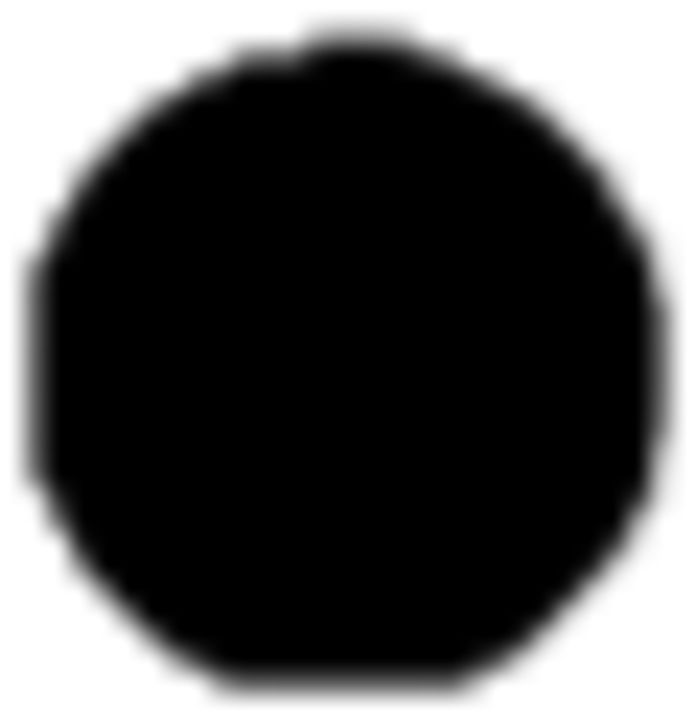	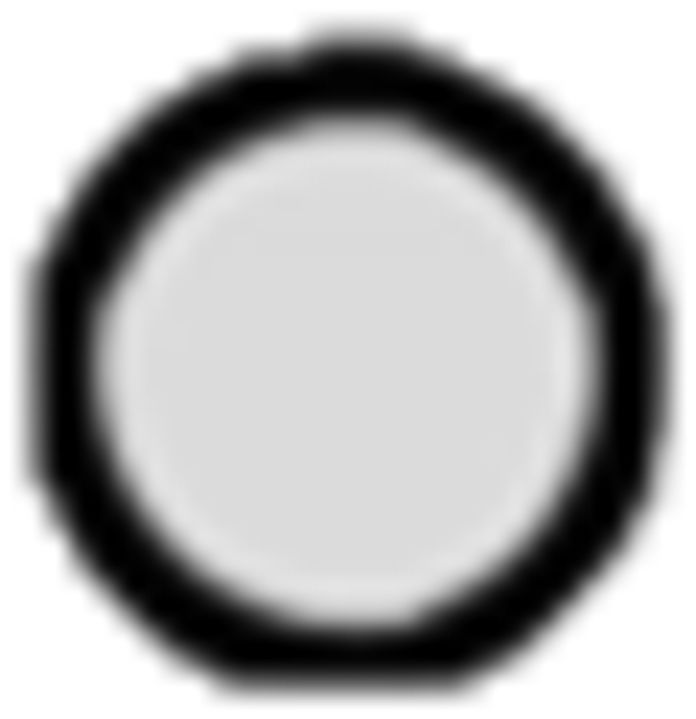	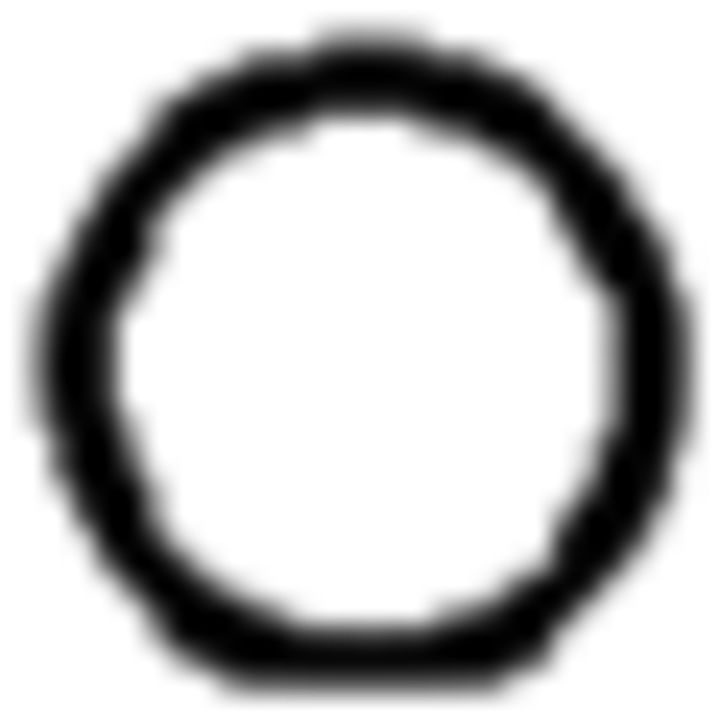
Time	The sense of time weakens as SB becomes more flexible, in the third stage, it eventually disappears. Specifically, we are referring to a sense of a past continuing into the future; to the reduction in the sense of duration; and finally, to the sense of continuity itself which also disintegrates. It seems that the sense of time is a “mirror reflection” of the SB. Thus, any alteration in the level of flexibility is reflected by an adjustment in the sense of time.	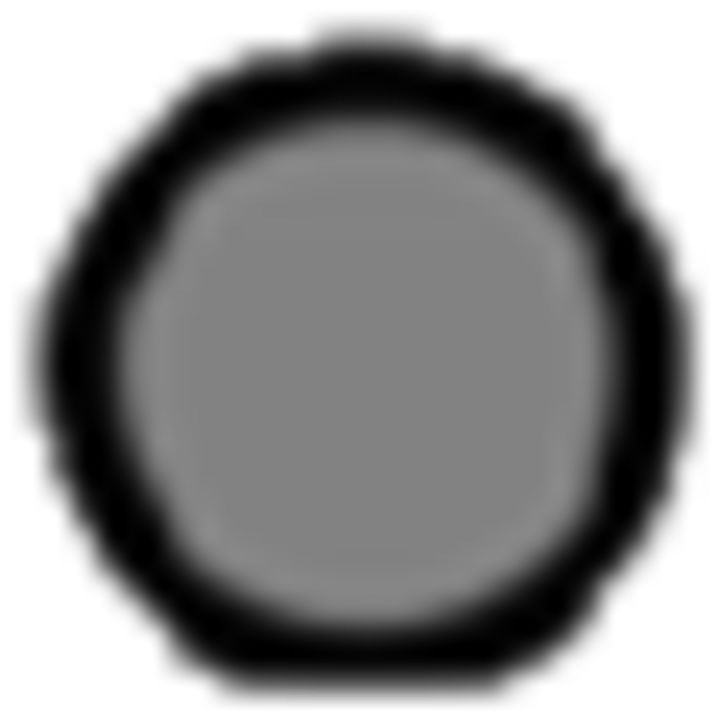	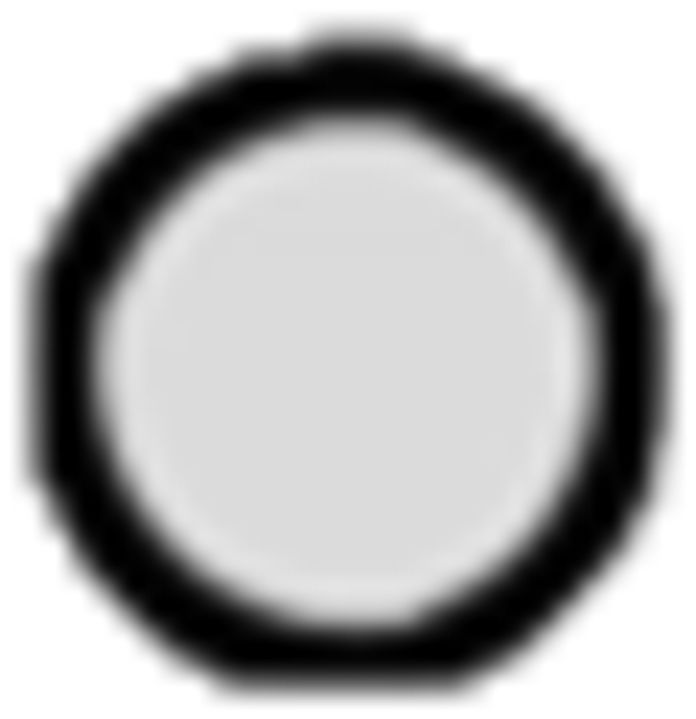	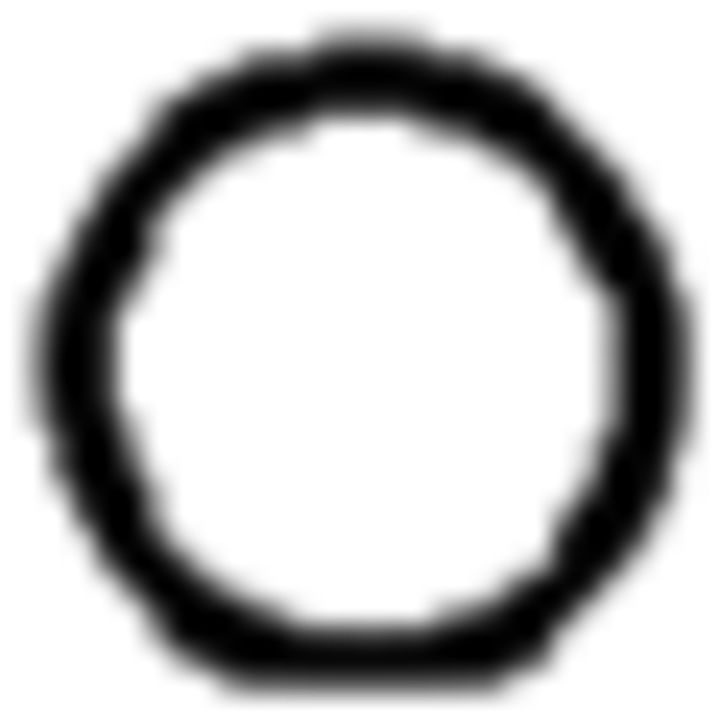
Location	As the SB becomes more flexible, one's ability to locate oneself in space deteriorates. The sense of location is always relative to objects in space. When the SB becomes more flexible, the intentional structure weakens and, in turn, objects become less distinguishable. The ability to locate oneself dwindles gradually: at first (SB2) the ability to differentiate between left/right and up/down decreases and, subsequently (SB3), the sense of orientation in space is lost altogether.	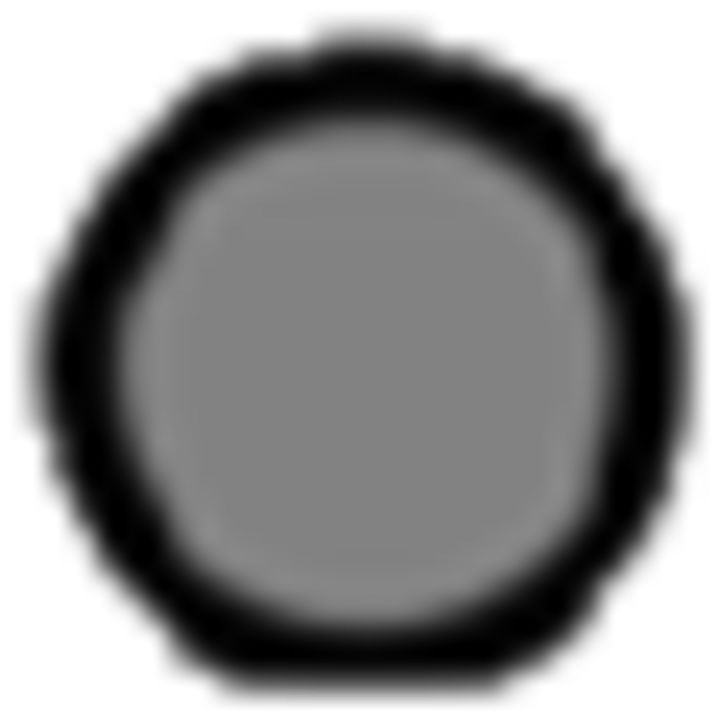	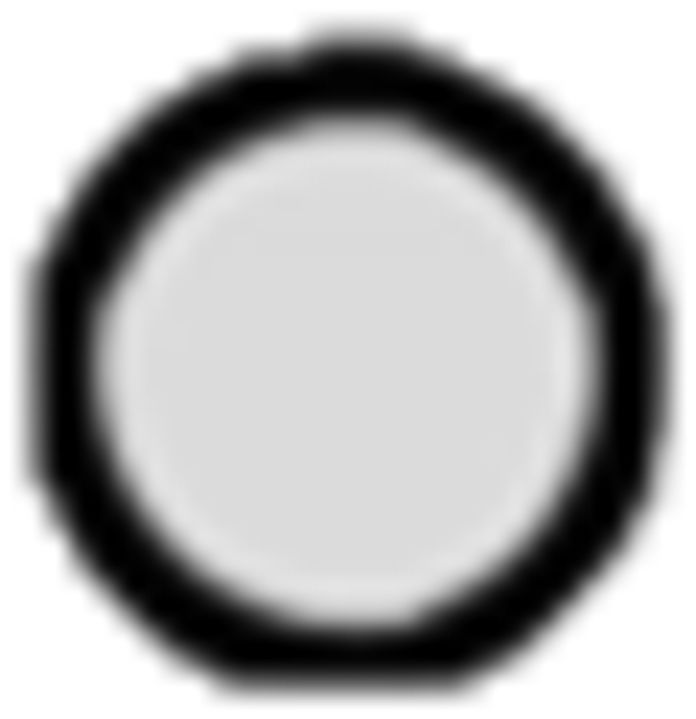	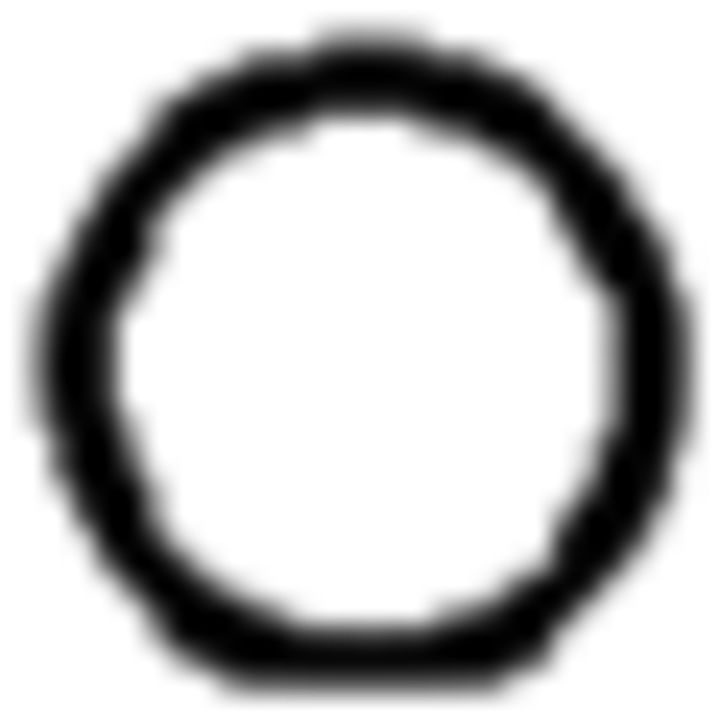
Self	As the SB becomes more flexible, the sense of self dissolves, thus becoming weaker. This process begins by expanding the sense of self (SB2) and, thereafter (SB3), as the SB disappears the sense of self disappears altogether.	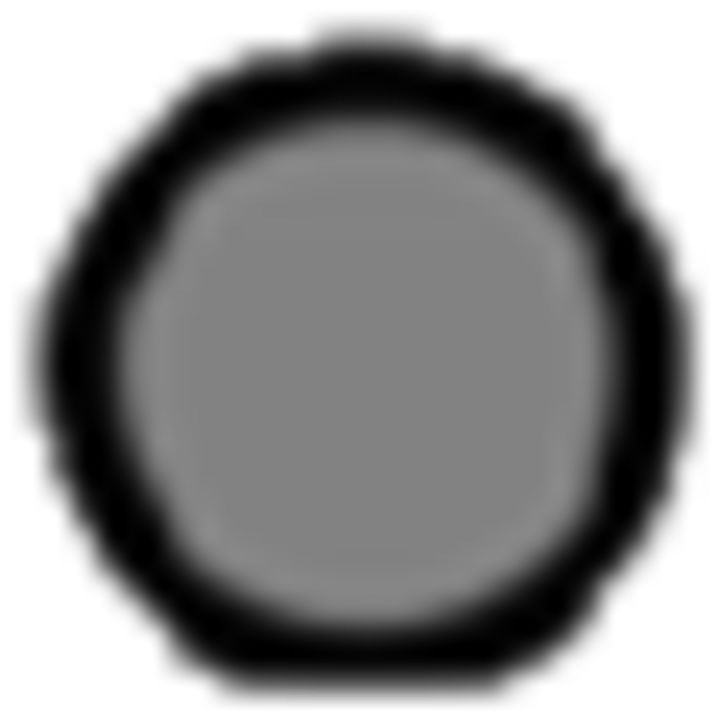	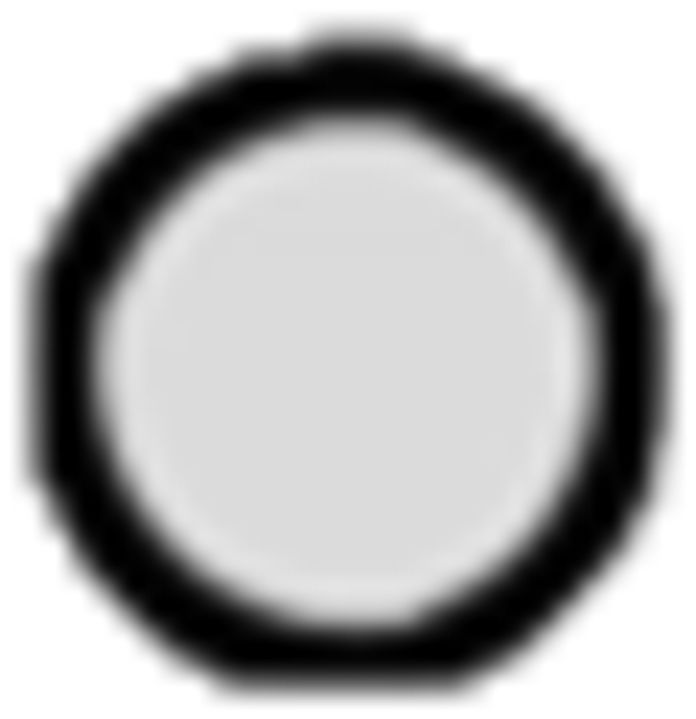	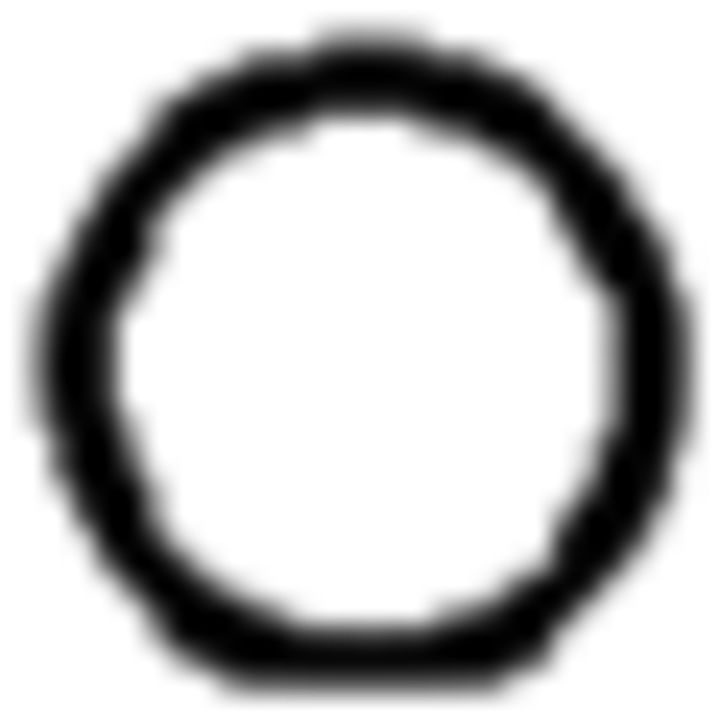
Agency	With an increase in the flexibility of the SB, the *need* for control declines. While in the SB2 the potential to act still exists, hence some sense of agency remains, in SB3 it disappears completely.	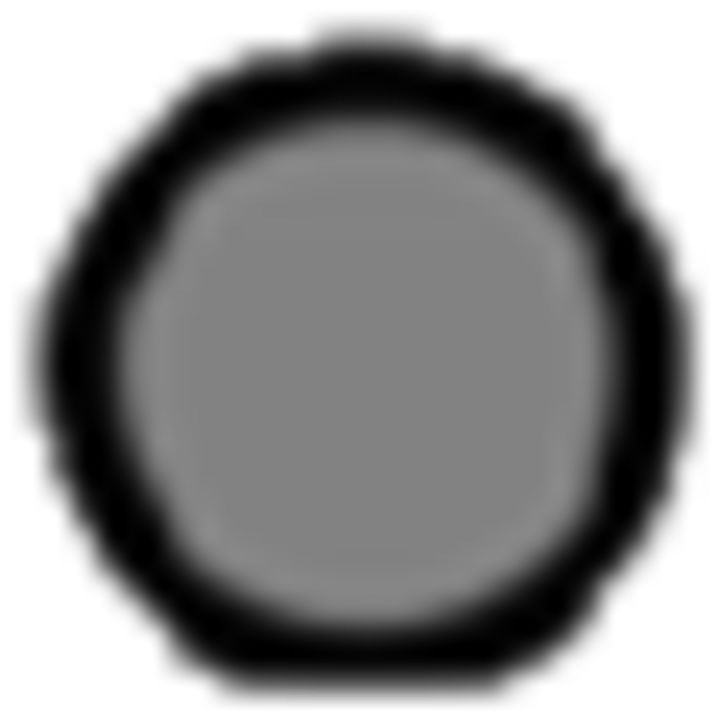	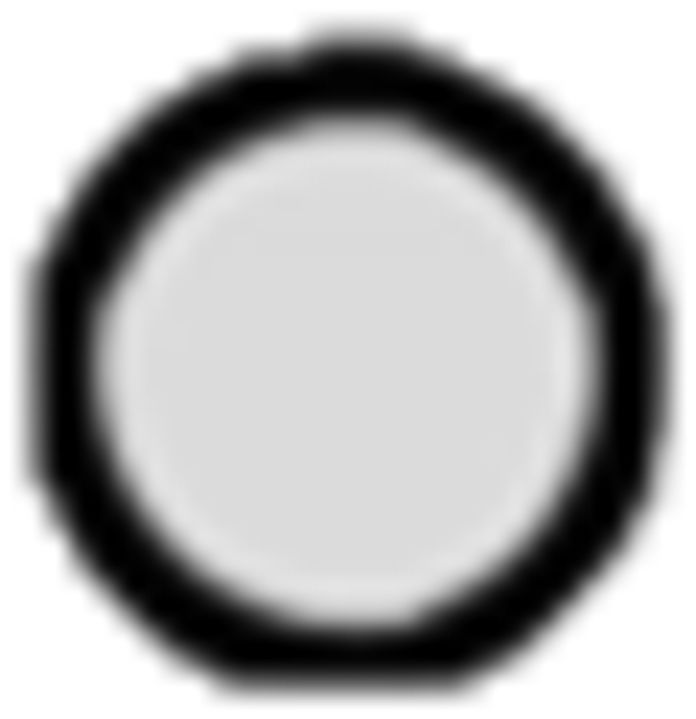	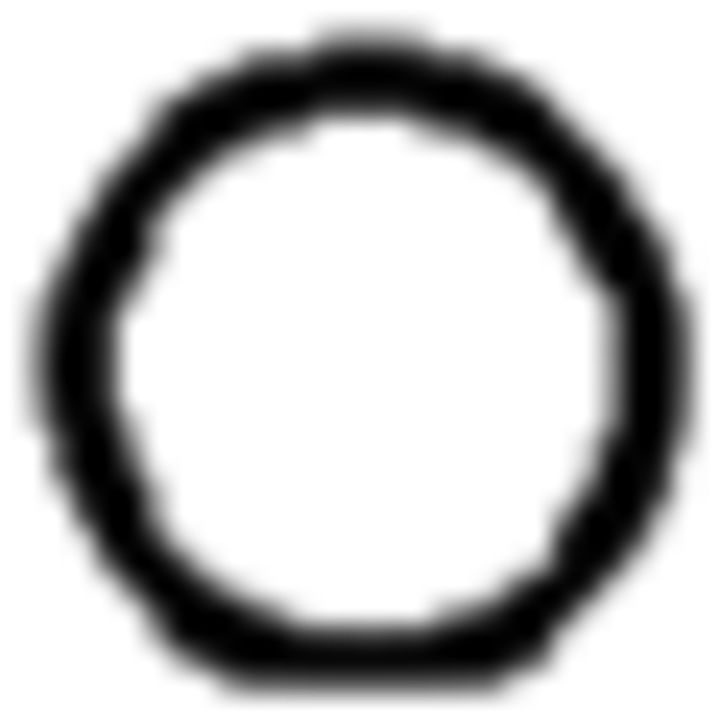
Ownership	As the SB becomes increasingly flexible, the sense of ownership (SO) becomes weaker. In the second stage a very thin SO remains, while in the third stage the SO disappears completely.	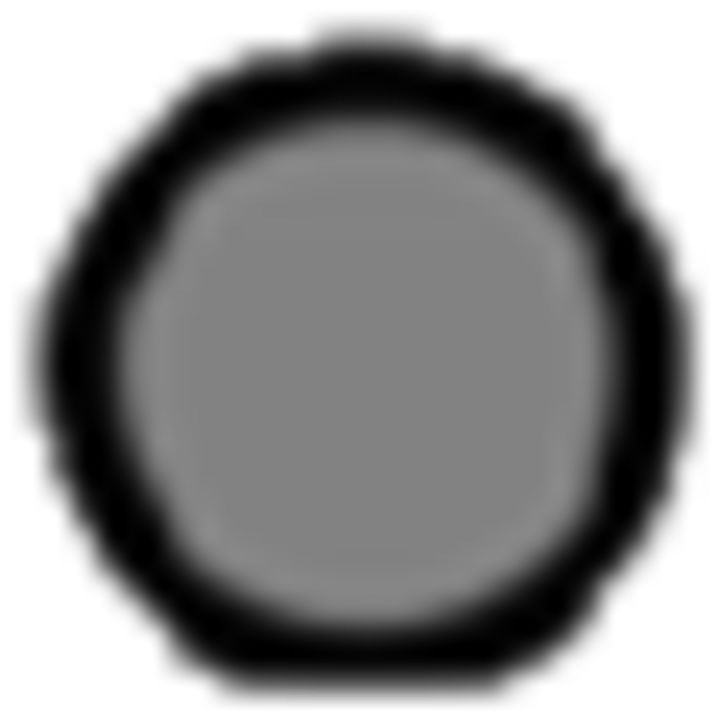	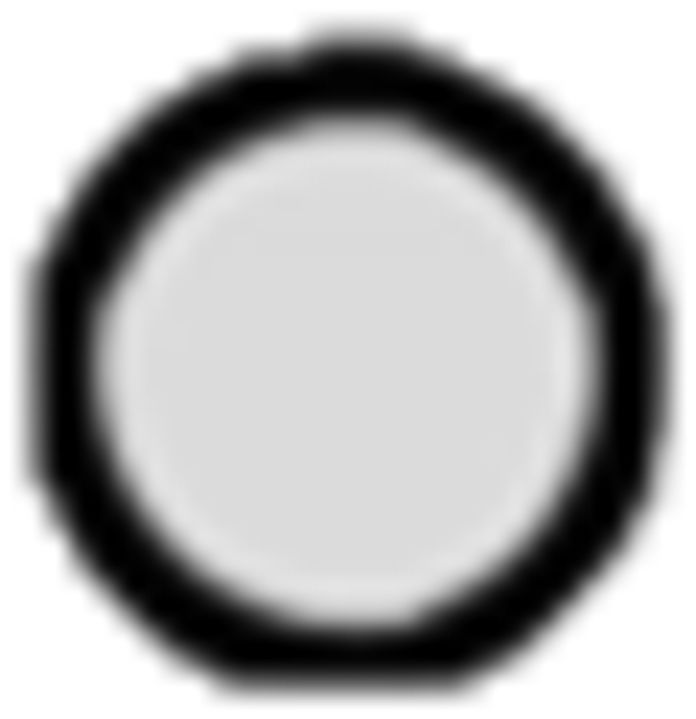	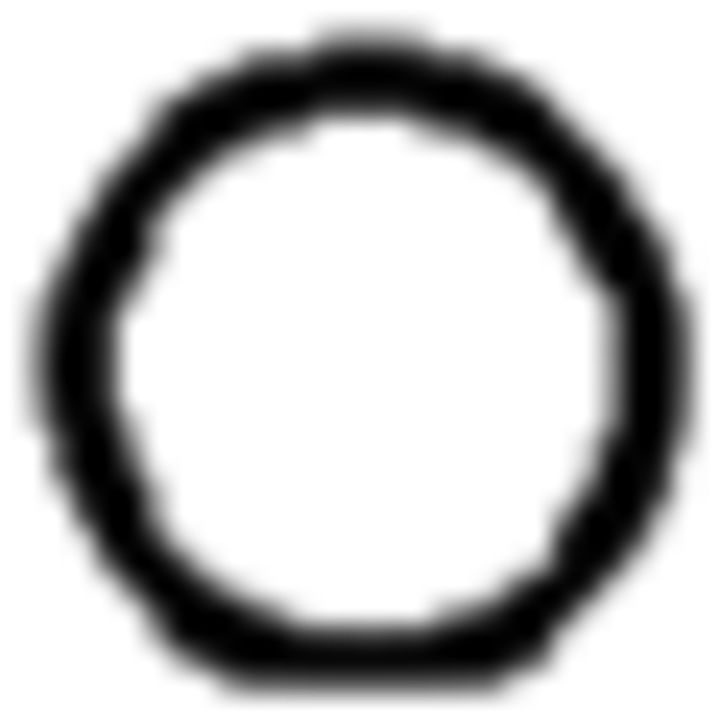
Center	First-person-egocentric-bodily perspective. As the flexibility of the SB increases, the sense of being at the center (with one's body as a reference point) deceases until eventually, in SB3, the body ceases to act as a reference point in relation to the outside world	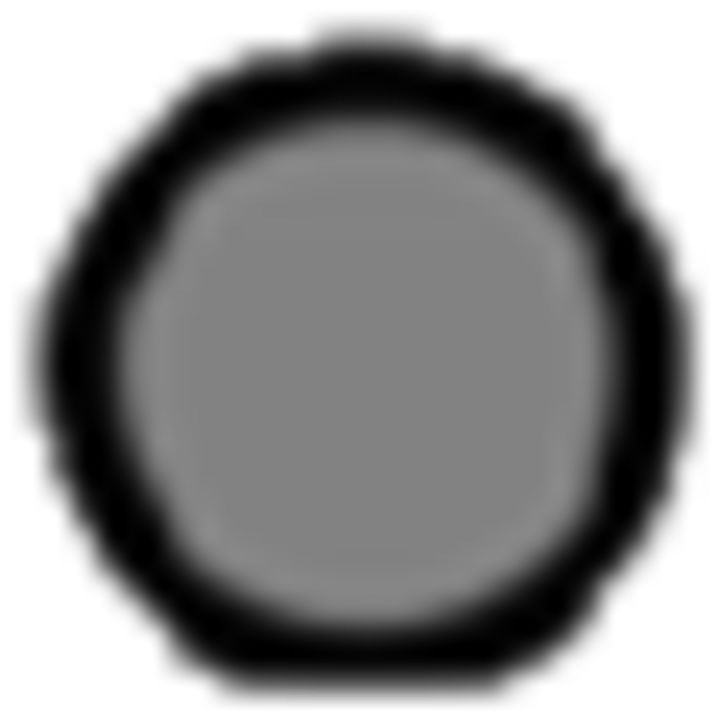	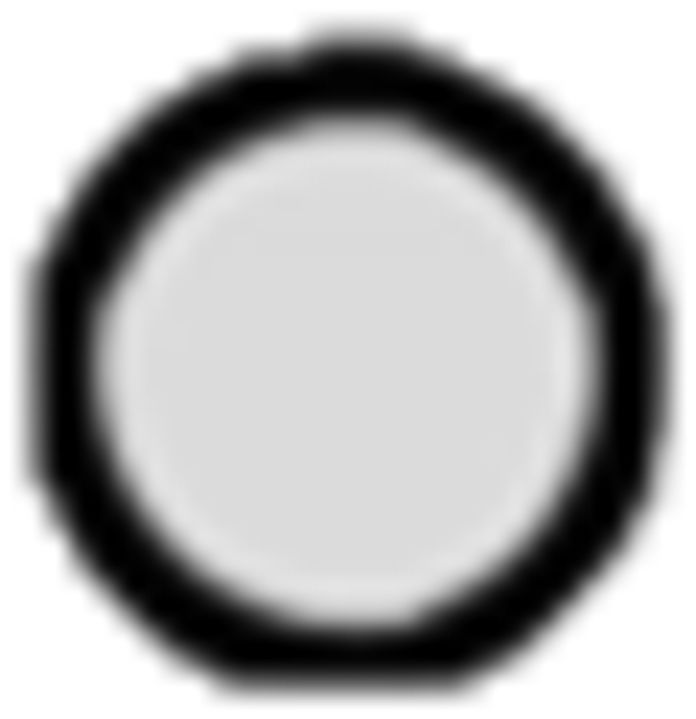	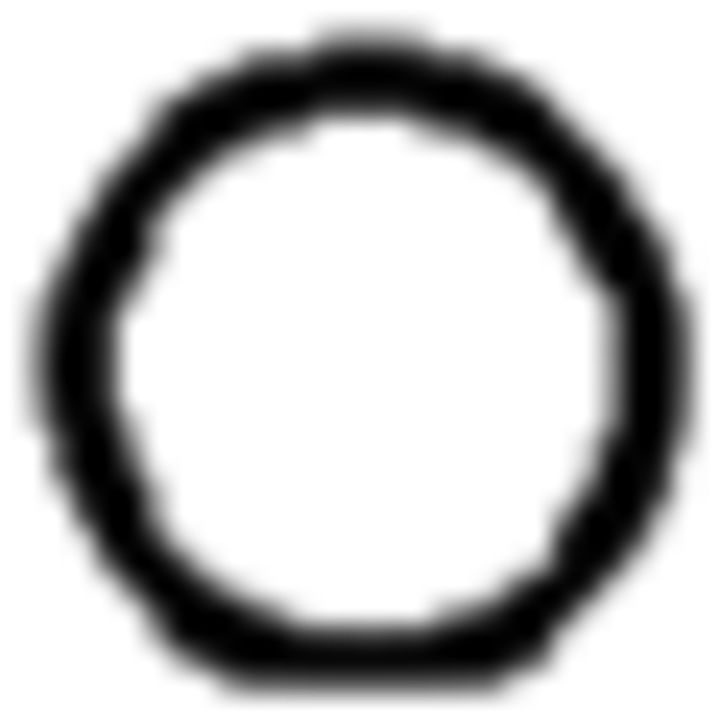
Touching-touched structure (TTS)	When touching an object, the boundary between subject and object is at its clearest. Essentially, the TTS stands at the core of the intentional structure. As the SB becomes increasingly flexible, the TTS weakens, yet “it does not disappear altogether.” One can undergo a very fluid touching/being-touched kind of experience without generating a SB. This notion is comprehensible when the TTS is described on the level of the entire body touching (and being touched) by the world.	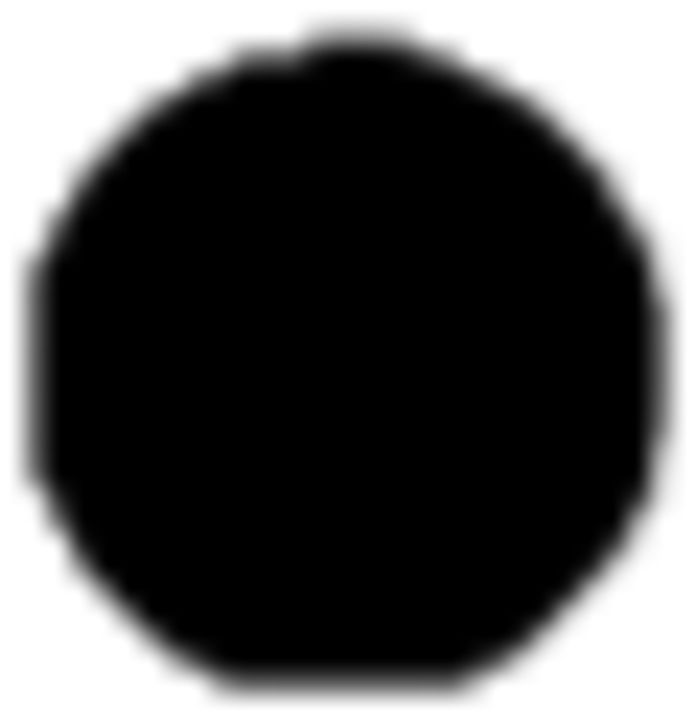	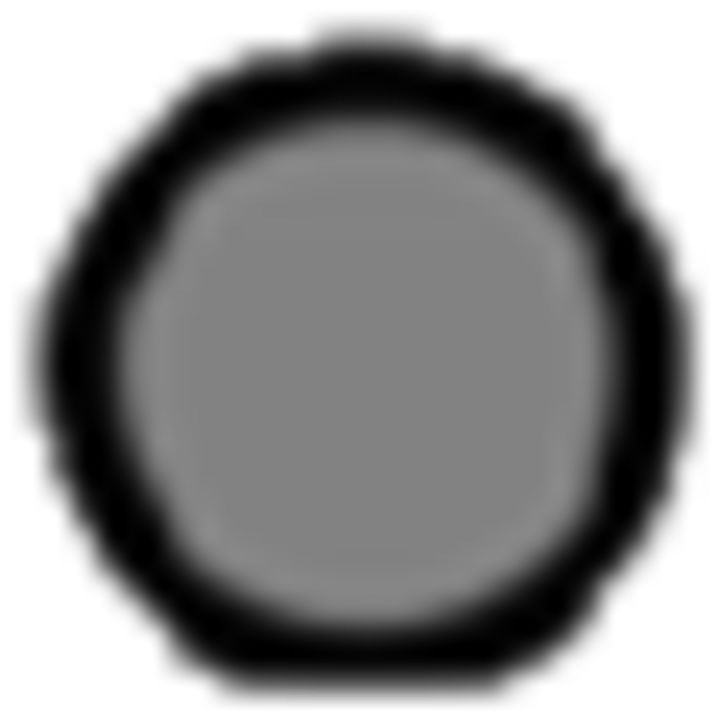	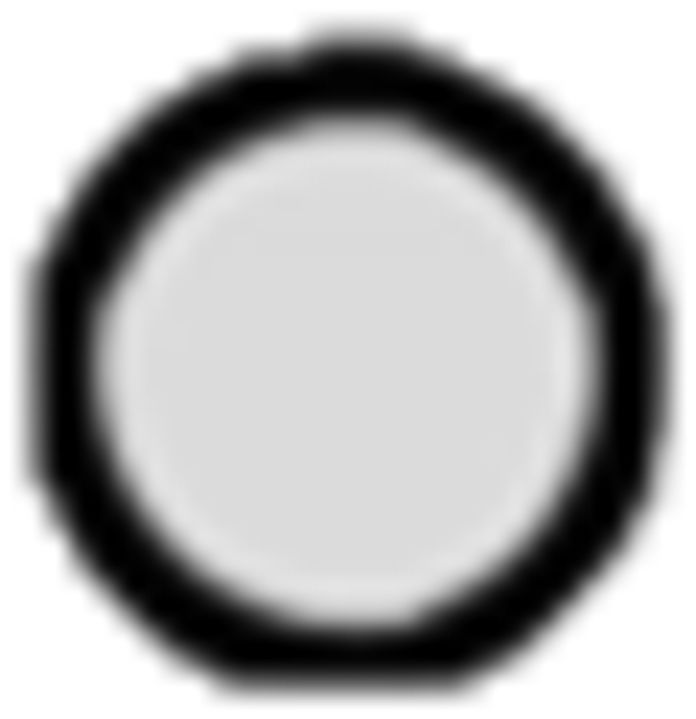
Bodily feelings	As the SB becomes increasingly flexible, bodily feelings, including proprioception and kinesthesia, become weaker. Yet even when the SB disappears, a minimal level of dynamic proprioception continues to exist: there remains a sense that there is a body without any experience of an SB	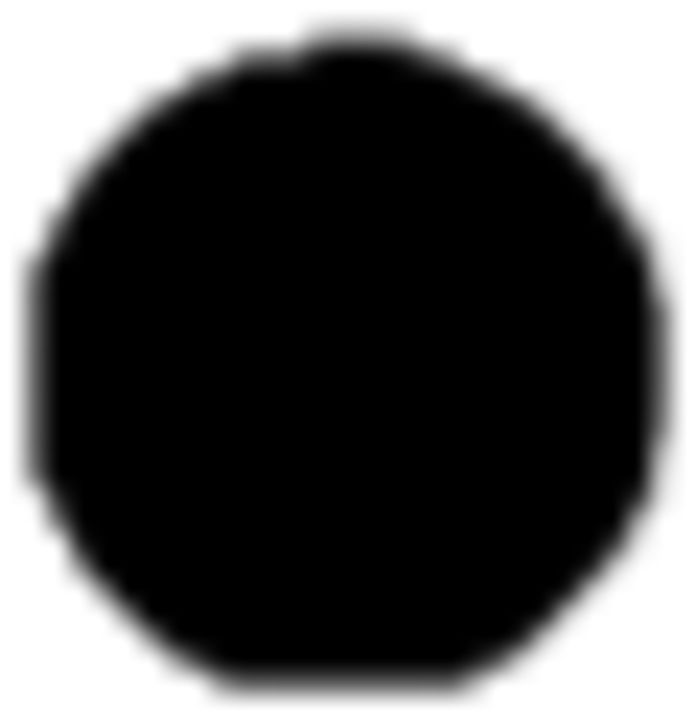	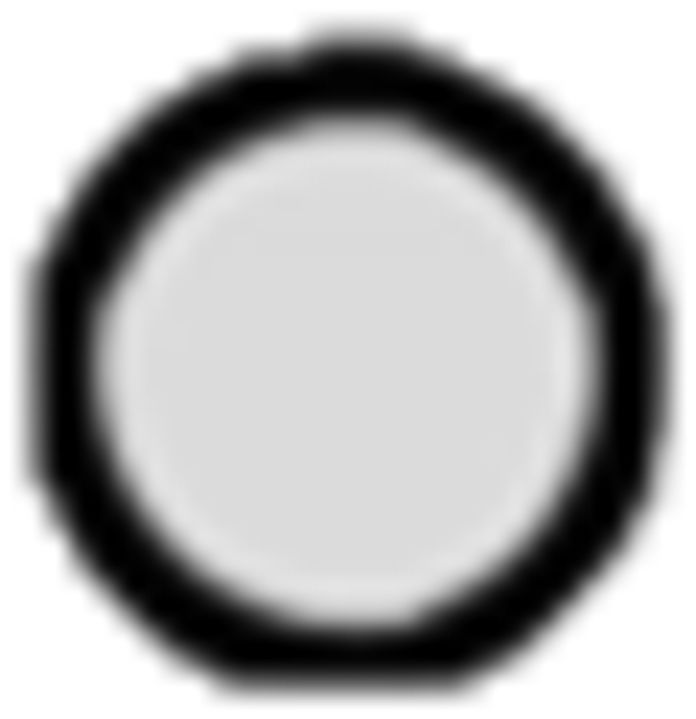	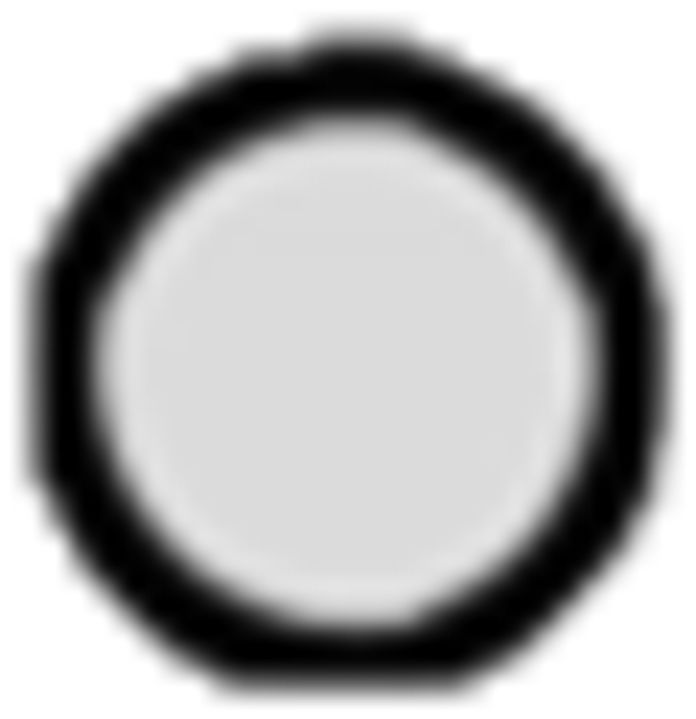

**Key:** 
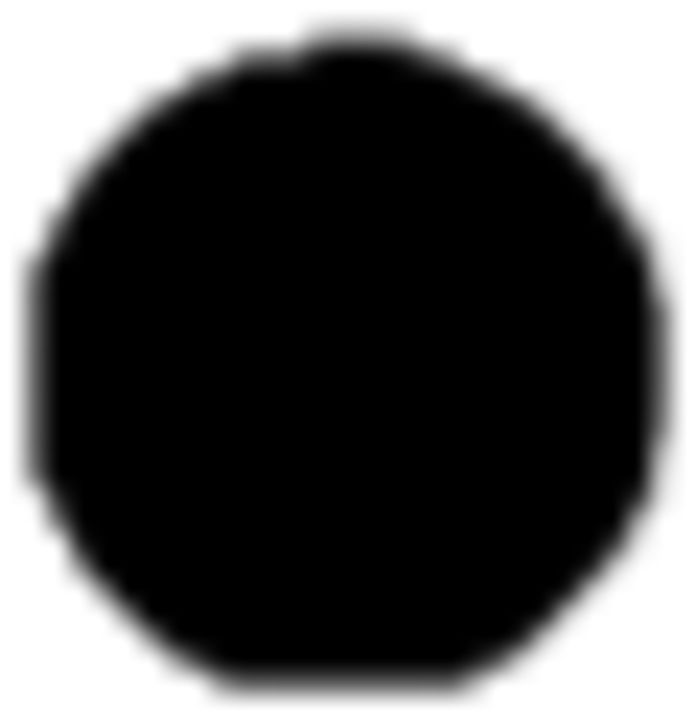
 strong; 
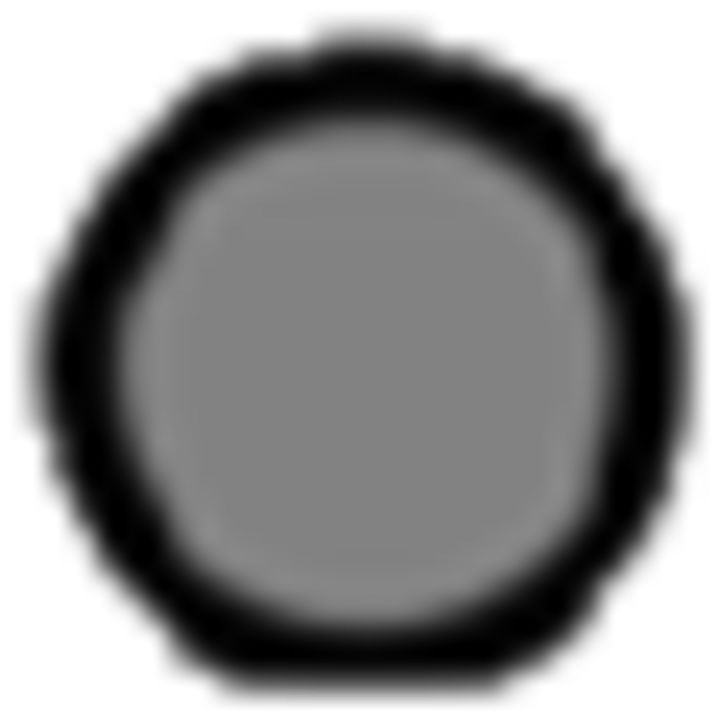
medium; 
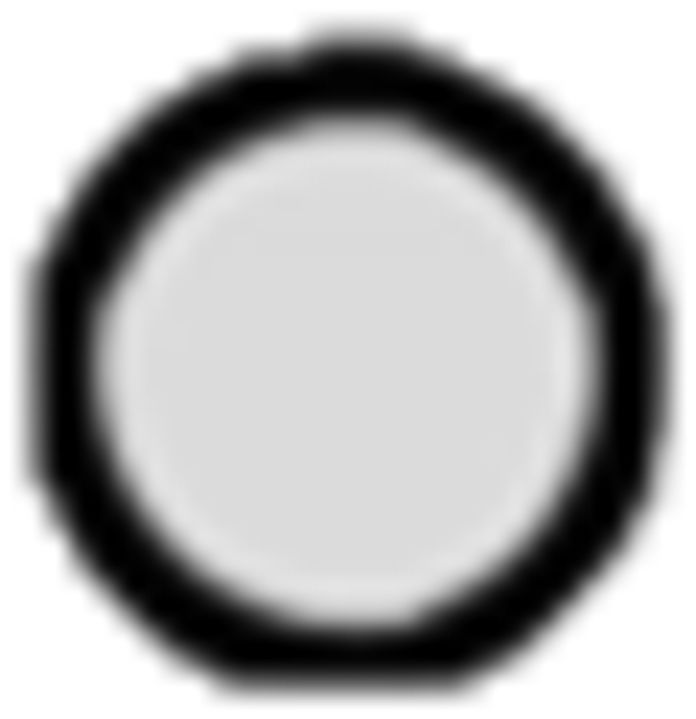
 weak; 
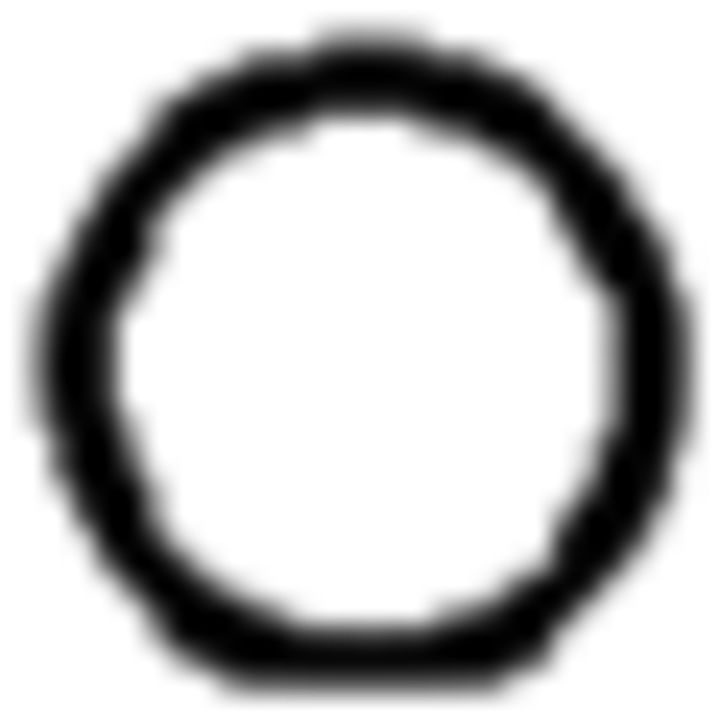
 nonexistent.

The phenomenal categories outlined above map onto theoretical and experimental conceptualizations which have been previously explored by phenomenologists and cognitive neuroscientists. Specifically, we are referring to self-awareness, including its extended/narrative and core/minimal aspects (using Damasio, 2010 and Gallagher, 2000 terminology, respectively). The categories of “time,” “location” and “self” map onto the self-as-object extended self-conceptualization (Damasio, 2010), whereas the core/minimal self-concept has been argued to be composed of the categories of “internal-external,” (Christoff *et al.*, 2011) “agency,” and “ownership” (Gallagher, 2000) and “center” (Zahavi, 2006). The status of the “TTS” and “bodily feelings” categories is less clear (Gallagher, 2000, 2013). Though possibly related to the minimal self-concept, they are better understood in terms of Damasio’s (2010) proto-self-concept, conceptualized as primordial feelings of the living body (such as proprioception and kinesthesia), which precede the subjective experience of being a self. The suggested pre-minimal-self-status of the “TTS” and “bodily feelings” agrees with the phenomenal results that these two categories remain, to some degree, even when the SB (as defined by seven of its categories) disappears. The close link between the nine phenomenal categories and the narrative/minimal/selfless modes of awareness are further clarified in the Supplementary Material (Section 1.1, Supplementary Fig. S1). As a final point, it is important to keep in mind that the different modes of self-awareness are not mutually exclusive. While not self-specific, SRP (such as the narrative mode) do include also SSPs. Like other conscious mental content produced by the brain, SRP content expressed as thoughts and feelings is stamped with the subjective signature of being our thoughts and feelings. Thus, an encapsulated working model of self-awareness modes has been suggested (Gallagher, 2000; Damasio, 2010; Dor-Ziderman *et al.*, 2013) and is adopted here.

These phenomenological insights were “front-loaded” (Gallagher and Sørensen, 2006) onto the experiment’s design and analysis in two ways. First, the gradually descending states of SB guided us toward performing a regression analysis, and thus examining not just differences between two brain states, but differences specifically related to the gradual process of SB dissolution. We thus searched for spatial and oscillatory, sensor and source-level signals which increased or decreased their activity together with the three different SB states. Second, the close phenomenological link between the experience of SB and self-awareness mode allowed testing the neural results on an independent, previously recorded MEG dataset of proficient contemplative practitioners (reported in Dor-Ziderman *et al.*, 2013), who produced in the MEG states of gradually descending self-awareness from an extended narrative sense of self to a minimal sense of self focused on the “here and now,” and finally to a selfless mode of awareness where the sense of ownership disappeared (see section “Methods” for more details). We hypothesized that: (i) The decrease in the SB would correlate with identifiable oscillatory systems in the brain (Stage 1), and that (ii) these would generalize to the meditator group’s data (Stage 2). We used MEG as the study’s research tool as it allows noninvasive but reliable source estimation of fast neural oscillatory rhythms (Hansen *et al.*, 2010).

## Methods

### Stage 1

#### Participant

We collaborated with S (third author of this article), a male aged 64, who has been practicing mindfulness according to the Satipatthana and Theravada Vipassana traditions for about 40 years, with over 20 000 accumulated hours. S was chosen for the present study for two reasons: (i) His proven skill (based on five previous phenomenological interviews) in producing on demand unique states of consciousness, sustaining them, describing them in rich detail as they unfold, carrying out reflexive processes without “interfering” with the first-person pre-reflexive experience, and precisely defining the limitations of his descriptions; (ii) His ability to accomplish these feats under experimental conditions. Laboratory settings introduce a set of nontrivial constraints and pressures which can make it difficult even for experienced practitioners to perform as well as they would under optimal conditions. Over the past 10 years, S has been collaborating with neuroscientists in neuroimaging studies using a variety of methodologies including electroencephalogram (EEG) (Berkovich-Ohana *et al.*, 2012, 2014), MEG (Berkovich-Ohana *et al.*, 2013; Dor-Ziderman *et al.*, 2013), and fMRI (Berkovich-Ohana *et al*., 2016). Regarding S’s involvement in the study, S’s role in the study was that of a unique subject who generously contributed his time, effort, and unique expertise to the practice, production, and description of the SB states in a manner conducive to neurophenomenological research. He was not involved in the formulation of hypotheses, analysis of the data (phenomenological and neurophysiological), or in the interpretation of the results.

#### Procedure

**Figure 1:**
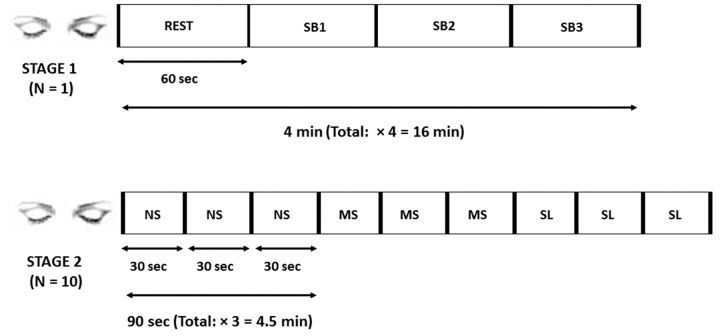
Experimental setup for Stage 1 (top) and Stage 2 (bottom). SB, sense of boundaries; NS, narrative self-mode; MS, minimal self-mode; SL, selfless mode. All epochs were initiated by an auditory cue.

Immediately after accepting S's approval, a series of meetings were set up in which the proposed study was introduced, the optimal number of SB states determined, and it was verified that S could produce on demand states corresponding to different levels of SB which were (i) replicable, (ii) differentiated enough, and yet (iii) true to the complexity of the experience, and finally, (iv) open to a high level of description. A phenomenological interview (following the methods described in Petitmengin, 2006 and Vermersch, 2009) took place in the MEG laboratory while lying supine within the scanner with closed eyes—in conditions similar to the subsequent brain recording session. S described his inner experience after meditating as well during the meditation itself. The full interview transcripts are available online as Supplementary Material to Ataria *et al.* (2015). The experimental design included four blocks, each block consisting of three 1-min conditions of normal SB (SB1), attenuated SB (SB2), and a state in which the SB disappeared completely (SB3). In between blocks there was a 1-min rest period which was not analyzed. The order of conditions remained constant throughout the blocks due to the difficulty in randomly producing the different states. S was cued by prerecorded aural instructions (the words “one,” “two,” “three,” and “rest”) to transition from one state to another (see Fig. 1). Though S reported he could transit almost instantaneously between states, the first 3 s of each block were not analyzed in order to ensure sufficient time for state stabilization.

#### MEG data acquisition

MEG recordings were conducted with a whole-head, 248-channel magnetometer array (4D Neuroimaging, Magnes 3600 WH) in a magnetically-shielded room. Reference coils located approximately 30 cm above the head oriented by the x, y, and z axes were used to remove environmental noise. Head position was indicated by attaching 5 coils to the scalp and determining, to a 1 mm resolution, their position relative to the sensor array before and after measurement. Discrepancies of less than 5 mm were measured in each of the five coils. Head shape and coil position were digitized using a Pollhemus FASTTRAK digitizer. Brain signals were recorded with a sample rate of 1017.25 Hz and an analog online 0.1–400 Hz band-pass filter. The instructions for each condition were presented using E-prime 2.0 and delivered via a STAX SRS-005 amplifier and SR-003 push-pull electrostatic earspeakers coupled with a vinyl tube to silicon earpieces to prevent magnetic noise within the shielded room.

#### Cleaning and preprocessing

Data processing and analysis was performed using Matlab®R2009b and FieldTrip toolbox for MEG analysis (Open Source Software for Advanced Analysis of MEG, Oostenveld *et al.*, 2011). Data were cleaned for line frequency (by recording on an additional channel the 50 Hz from the power outlet, and subtracting the average power-line response from every MEG sensor), building vibration (measured in x, y, and z directions using three Bruel and Kjaer accelerometers), and heartbeat artifacts using the methods described in Tal and Abeles (2013). One malfunctioning MEG sensor was identified and its data were removed from further analysis. The data were segmented into nonoverlapping 2-s epochs, which were visually examined for muscle and jump (in the MEG sensors) artifacts. Contaminated epochs were removed from further analysis. To ensure the removal of all heartbeat, eye, and muscle artifact, an independent component analysis (ICA) was performed on the data (Jung *et al.*, 2000). Segmented data were down-sampled to 300 Hz to speed up data decomposition. The data were then decomposed into a set of independent components (247, as the number of remaining sensors) ordered by degree of their explained variance. Components indicating heartbeats or eye movements were determined by visual inspection of the 2D scalp maps and time course of each component. Two components were taken out, and the resultant unmixing matrix was used to compute the time-courses of the data in its original high-temporal resolution.

#### Sensor space analysis

A whole-head frequency analysis for low (2–40 Hz) and high (40–90 Hz) frequencies at 1 Hz resolution was performed. For each 2-s epoch in each sensor, a Fast Fourier Transform (FFT) with a Hanning taper was applied to overlapping fixed-length sliding time windows (every 50 ms), which were then averaged, yielding one value per sensor, epoch, and frequency. Fixed-time windows of different lengths as well as different degrees of spectral smoothing were used for low and high frequencies: 0.5 s and 2 Hz smoothing for 2–40 Hz, and 0.2 s and 14 Hz smoothing for 40–90 Hz (Gross *et al.*, 2013).

For determining frequency-specific neural activity related to the gradual decrease in the sense of boundaries (SB), the epochs were grouped into the three conditions outlined previously (SB1, SB2, SB3) and subjected to a regression analysis, yielding a regression coefficient *t*-statistic. This procedure was performed independently (over sensors/frequencies) in two stages (see Levy *et al.*, 2013; Van Der Werf *et al.*, 2010 for a similar analysis strategy): initially, after averaging over all the sensors, thus determining frequency of interest (FOI). Statistical results of this stage were corrected for multiple comparisons by controlling the false discovery rate (FDR, Benjamini and Hochberg, 1995). Once statistically significant FOI’s were determined, the regression procedure was performed for each sensor individually averaged over the FOI, thus determining the sensors of interest (SOI) driving the FOI effects. Statistics were assessed using a cluster-based nonparametric permutation approach on pooled regression *t*-values (Maris and Oostenveld, 2007). Finally, the results were collapsed across the SOI within the FOI range to yield one value per trial per condition, and post hoc *t-tests* were conducted and Bonferroni corrected.

One drawback of the study’s setup is that the order of the SB states was fixed (see Fig. 1). This design was adopted as a completely random ordering of conditions introduces further difficulty in producing the SB states. However, as in each of the four blocks, SB1 preceded SB2 which preceded SB3, it could be argued that regression results could be due to temporal order and not due to the nature of the SB states themselves. In order to rule out such an explanation, the data was regrouped into conditions based on temporal order alone (order of presentation), and an identical regression analysis was performed on the newly-grouped data (see Supplementary Fig. 3 in Section 2.1.1). For ascertaining that the reported power changes were indeed increases/decreases in power rather than reflecting a return to equilibrium, an additional control measure was implemented. The 1-min rest periods in each block were used for baseline correction of the SB data (see Supplementary Section 2.1.2 and Supplementary Fig. 4).

#### Source space analysis

Neuronal sources which could account for the significant sensor-level results were estimated using an adaptive spatial filtering method (beamforming, Gross *et al.*, 2001), relying on partial canonical correlations. Spatial filters optimally pass activity from the location of interest, while suppressing activity from other locations. The subject's brain volume was divided into a regular 1 cm grid and aligned to a template brain (Montreal Neurological Institute, MNI), with the positions determined by linear transformation from an equally sized canonical grid based on the template brain. Lead field matrices were computed using a single-shell volume conduction model (Nolte, 2003) based on the manually-digitized headshape. This process was performed with matlab scripts using SPM8 (http://www.fil.ion.ucl.ac.uk/spm, last accessed 9 March 2016).

Fourier transforms of the tapered data epochs were computed for the frequency bin that yielded the most extreme *t*-value in the sensor analysis, using a Hanning window and 4 Hz spectral smoothing. Spatial filters were then constructed for each grid location (using all three conditions), and the data were projected through the spatial filters and log-transformed. This procedure yielded source-level power estimates for each epoch, condition, and voxel. The data in each voxel were then subjected to the same statistical regression analysis performed at the sensor level, and the whole-head image was again corrected for multiple comparisons using cluster-based permutations. For the purpose of using the visualization options and anatomical atlases afforded by the AFNI-SUMA software (http://afni.nimh.nih.gov/afni, last accessed 9 March 2016), the final statistical images were transformed to Talairach space (Talairach and Tournoux, 1988).

### Stage 2

#### Setup

The participant group consisted of 12 right-handed long-term mindfulness meditators (9 males and 3 females, mean age 45.2, averaging over 16 years and 11 000 h of formal practice). This group included S as it was part of a larger experiment containing tasks unrelated to the ones reported here (some of them reported in Berkovich-Ohana *et al.*, 2013; Dor-Ziderman *et al.*, 2013). S’s data, however, was not analyzed to avoid confirmation circularity. Here we used the latter dataset but implemented a novel analysis procedure, matching the current study’s aims. The purpose of the previous study (Dor-Ziderman *et al.*, 2013) was to outline the differential spatial and spectral mechanisms mediating narrative versus minimal modes of self-awareness. The current analysis set out to partially validate Stage 1 results on an independent group of meditators by determining whether the hypothesized brain mechanisms (regions and frequency bands) were indeed correlated with the subjective attenuation of self-awareness.

As part of the experimental procedure, the participants were requested via aural prerecorded instructions to produce, volitionally, three modes of awareness for 30 s, three times for each state with their eyes closed (see Fig. 1). The first mode was “narrative” (operationalized as *“*try to think what characterizes you*”*), defined as a mode of self-awareness weaving episodic memory, future planning, and self-evaluation together with a coherent self-narrative and identity; the second was “minimal” (operationalized as “Try to experience what is happening to you at the present moment”), a minimal mode of self-awareness focused on present momentary experience and closely tied to the sense of agency and ownership (Gallagher, 2000); and the third was “selfless” (operationalized as “Try to experience what is happening at the present moment, when you are not in the center”), a mode of awareness defined by and practiced within Buddhist contemplative traditions, in which identification with a static self is replaced by identification with the phenomenon of experiencing itself (Dalai Lama, 1997). As this mode of awareness may be alien to readers unfamiliar with meditation experience, we supply below two of the meditators’ experience reports: sub12: “It was emptiness, as if the self fell out of the picture. There was an experience but it had no address, it was not attached to a center or subject. It was not 100%, but there was no sense of an object there running the show.”; and sub14: “It was to be aware of the body, the sensations, pulse, location of limbs, sounds and sights—to be only a witness to all this.”

Throughout the successive volitional shift of the sense of self between the three modes, MEG was recorded. Online as well post-experiment retrospective data were collected including measures of task success and stability. The online measure was a 1–3 (3 meaning no success) rating of task performance success after each 30-s epoch (triggered by a bell sound with 3 indicating no success). No scores of 3 were recorded. The retrospective measures were collected outside the MEG after the experiment. The scores were high: an average of 8.13 and 7.93 (on a 1–10 scale) for success and stability, and were not significantly different across the narrative, minimal, and selfless conditions (for further details, see Dor-Ziderman *et al.*, 2013). Regarding first-person data, as talking during MEG recording may cause movement artifacts, the collected phenomenological descriptions were limited to the selfless state which was the last state produced (see Fig. 1). Immediately after participants produced this state, the MEG recording stopped and first-person descriptions were collected.

#### Analysis and statistics

The details regarding MEG data collection, cleaning, and preprocessing are similar to what has been described in Stage 1. The data of one subject could not be analyzed due to a volume alignment problem. The data of the remaining 10 subjects were analyzed in two stages: First, we checked whether significant regression values were present for the delta (1–4 Hz), theta (5–7 Hz), alpha (8–13 Hz), beta (14**–**30 Hz), low gamma (31**–**50 Hz), and high gamma (51**–**90 Hz) frequency bands. This was done by averaging over all sensors for each subject and then conducting a one-sample t-test of the pooled group results against the null hypothesis that the distribution would have a zero mean. We hypothesized that significant frequency band/s would match those found in Stage 1. In order to control for carry over effects possibly resulting from the lack of counterbalancing or randomization of the trial/block type, the same analysis was performed on two other conditions (related to the sense of “time” and “space”) which were identical in design (three states, each state produced for 3 x 30 s, see Supplementary Fig. S4 for more detail). We hypothesized that here the significant frequency band/s would not match those found in Stage 1.

In the second stage, source localization was carried out in a similar manner to the one described in Stage 1. For facilitating a group analysis, all brain volumes were aligned to the same template brain detailed in the “Methods” section, thus creating a common anatomical space on which group statistics could be performed. Source estimation was computed on the peak frequency within the determined frequency band of interest from the Stage 2 group data. In addition, a one sample *t-test* of the Stage 2 group results against subject S’s results from Stage 1 was conducted in order to highlight differences in terms of the effects’ spatial overlap extent. Given that subject S is such a highly-skilled practitioner, we hypothesized that the effects found for S would also be more pronounced relative to the other meditators. Statistics were assessed using a cluster-based nonparametric permutation approach on pooled regression *t*-values (Maris and Oostenveld, 2007).

## Results

### Stage 1

#### Sensor space

**Figure 2:**
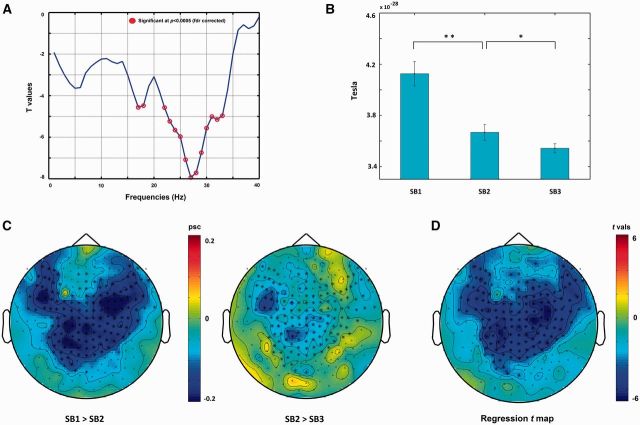
Sensor-level results. Determining FOI: (a) Frequencies (*x*-axis) regression *t*-values (*y*-axis) plot, averaged overall sensors. Red circles indicate statistically significant *t*-values (*P*<0.0005, FDR corrected); Statistical bar plot: (b) Mean power (*y* axis) and standard error bars averaged over FOI (22-33 Hz) and SOI for SB1, SB2, and SB3; Raw effect: (c) of percent-in-signal-change (psc) between SB1 and SB2 (left) and SB2 and SB3 (right). Color bar indicates psc from 0.2 (dark red) to –0.2 (dark blue); Determining SOI*:* (d) 2D regression *t*-map averaged over the FOI (22–33 Hz). Dots on the map represent sensors; stars signify significant sensors (*P*<0.0005, Monte Carlo permutation corrected). Color bar scale indicates *t*-values from 0.6 (dark red) to –0.6 (dark blue).* *P*<0.0335; ** *P*<1.07 x 10^**−7**^ (both Bonferroni corrected).

Averaging overall channels, the regression analysis indicated significant negative regression coefficient values in the beta band (marked by red circles), peaking at 27 Hz (Fig. 2a). No positive regression values were found in any of the frequency bands. In addition, regrouping of the conditions by order of presentation did not change the reported findings (see Supplementary Fig. 3), thus partially controlling for the fixed order of SB states production and ruling out the possibility that the results could be explained by their temporal ordering alone. In addition, the baselining of the data using the 1-min rest period in each block did not change the results meaningfully (see Supplementary Fig. 4), suggesting the results reflect decreases in beta band power (rather than a return to equilibrium).

The significant frequencies (22–33 Hz) were defined as FOI, interpreted as a beta band effect. While frequencies greater than 30 Hz are often interpreted as indicating the gamma band, the present data set suggests a beta band label to be more adequate. As can be seen in Fig. 2a, immediately after the 27 Hz peak, there is a sharp decrease in regression significance which continues and is maintained throughout the gamma band. In addition, significant peaks are present also in lower beta frequencies (16–18 Hz). These suggest that the spill into the higher frequencies reflects spectral leakage. Finally, the Stage 2 results (see section “Sensor space”), defined using a standard band definition (14–30 Hz), implicate the beta band (and only the beta band), further strengthening beta band interpretation of the results.

Subsequently, the effect’s spatial topography was examined. The spatial topography driving the beta band effect is presented in Fig. 2c and d, with significant sensors marked with bold stars. As can be seen the effect is pronounced over bilateral frontal and central sites. Fig. 2c shows the respective decrease in beta power percent in signal change (PSC) over these sensors; and Fig. 2d shows the overall statistical regression map. The mean power values over significant bands and sensors were averaged and subjected to a post hoc analysis (Fig. 2b).

#### Source space

**Figure 3:**
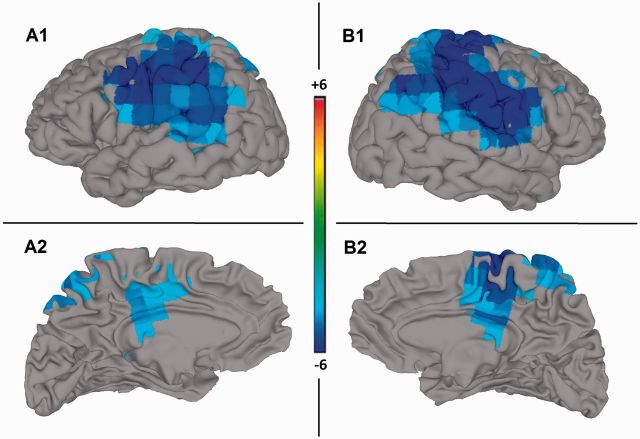
Beamforming beta band source estimation statistical images for subject S. Lateral (A1 and B1) and medial (A2 and B2), left (A1 and A2) and right (B1 and B2), views of S’s source estimates overlaid on SUMA 3D cortical surface model. Color bar indicates *t*-value degree from 6 (dark red) indicating a positive linear pattern to − 6 (dark blue) indicating a negative linear pattern. Images significant at *P*<0.0005 (Monte Carlo Permutation corrected). Lateral views (top) highlight the TPJ regions in both hemispheres; while the medial views (bottom) highlights the Prc and M/PCC gyrus bilaterally, and the SMA in the right hemisphere. For more detailed anatomical information, refer to Table 2.

The sources of the significant beta band regression for subject S are presented in Fig. 3, and further anatomical detail of the images is supplied in Table 2. In line with the sensor space data, only negative regression values were found. The results indicate a large widespread cluster of voxels manifesting over lateral (top images) and medial (bottom images) parietal regions, more extensively in the right hemisphere (right images). On the lateral surface of both hemispheres, the regions comprising the TPJ, namely, the inferior parietal lobule (IPL), supramarginal gyrus (SMG), angular gyrus (AG), superior temporal gyrus (STG), and middle temporal gyrus (MTG) are the main loci of the regression effect. In addition, the effect extends to primary sensory and motor regions and insular regions. On the medial surface, the precuneus (Prc) and middle/posterior cingulate gyrus bilaterally (M/PCC), as well as the supplementary motor area (SMA) in the right hemisphere also evidenced significant regression values.

**Table 2:** Beamforming beta band source estimation info for subject S (*n* = 1)

			Brain regions (Talairach–Tournoux atlas)	Overlap (%)	
				Left	Right
Total number of voxels (10 mm^2^)		329	Inferior Parietal lobule	5.4	6.1
			Postcentral gyrus	5.5	5.8
			Precentral gyrus	5.6	5.5
Hemispheric overlap			Cingulate gyrus	3.0	3.9
Left		Right	Superior Temporal gyrus	3.6	2.4
	36.4%	46.6%	Insula	1.4	1.9
			Supramarginal gyrus	1.8	1.8
Peak voxel			Medial Frontal gyrus(SMA)	———–	1.6
TLRC (mm, LPI)			Paracentral lobule	0.4	1.3
X	Y	Z	Middle Temporal gyrus	1.1	———–
40	−31	57	Middle Frontal gyrus	0.5	1.1
Located in the *right postcentral gyrus*			Precuneus	1.0	1.0
			Inferior Frontal gyrus	0.4	0.9
			Angular Gyrus	———–	0.8
Image threshold: p < 0.0005 (Monte Carlo permutation corrected)					

Information supplied includes total number of voxels, hemispheric overlap, peak voxel characteristics, image statistical threshold, brain regions involved, and their overlap with the significant voxels. The AFNI supplied TT Daemon atlas was used. Due to poor resolution and signal leakage to non-brain regions, overlap percentages do not add up to 100%.

**Table 3:** Beamforming beta band source estimation info for meditators group (*n* = 10)

			Brain regions (Talairach–Tournoux atlas)	Overlap (%)	
				Left	Right
Total number of voxels (10 mm^2^)		93	Inferior parietal lobule	———–	19.2
			Postcentral gyrus	———–	13.8
			Precentral gyrus	———–	7.2
Peak voxel			Supramarginal gyrus	———–	5.5
TLRC (mm, LPI)			Superior temporal gyrus	———–	3.2
*X*	*Y*	*Z*	Cingulate gyrus	———–	3.1
40	−62	31	Angular gyrus	———–	2.9
Located in the right			Superior parietal lobule	———–	2.4
angular gyrus			Insula	———–	2.3
			Inferior frontal gyrus	———–	2.3
			Precuneus	———–	2.3
Image threshold: *P*<0.0005 (Monte Carlo permutation corrected)					

Information supplied includes total number of voxels, hemispheric overlap, peak voxel characteristics, image statistical threshold, brain regions involved, and their overlap with the significant voxels. The AFNI supplied TT Daemon atlas was used. Due to poor resolution and signal leakage to non-brain regions, overlap percentages do not add up to 100%.

### Stage 2

#### Sensor space

The regression analyses over all sensors for the delta, theta, alpha, beta, low and high gamma bands yielded significant negative regression coefficient values in the beta band alone [T (1, 9) = −3.91, *P* < 0.0036 (0.018 after Bonferroni correction)], with the peak frequency at 21 Hz. The other frequency bands did not exhibit significant values (even before the Bonferroni correction). In addition, the regression analyses of the control “time” and “space” blocks in the beta band did not yield significant results, suggesting that the reported beta band effect could not be attributed to the fixed order of the blocks.

#### Source space

**Figure 4:**
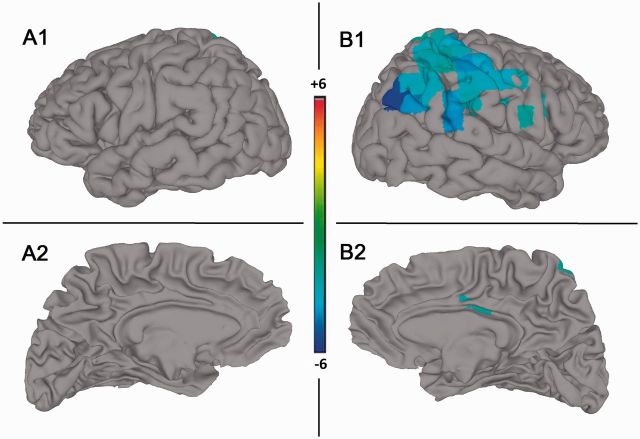
Beamforming beta band source estimation statistical images for meditators group (*n* = 10). Lateral (A1 and B1) and medial (A2 and B2), left (A1 and A2) and right (B1 and B2), views of the meditators group (*n*=10) source estimates overlaid on SUMA 3D cortical surface model. Color bar indicates *t*-value degree from 6 (dark red) indicating a positive linear pattern to − 6 (dark blue) indicating a negative linear pattern. Images significant at *P*<0.0005 (Monte Carlo permutation corrected). No results were found in the left hemisphere (A1 and A2). Right lateral view (B1) highlights the TPJ region; while the right medial view (B2) highlights the M/PCC and the Prc. For more detailed anatomical information, refer to Table 3.

The sources of the significant beta band regression for the group-level analysis (*n* = 10), masked by S’s ROI’s, are presented in Fig. 4, and further anatomical details of the images are supplied in Table 3. Again, only negative regression coefficients were found. The findings indicate a large right hemisphere cluster peaking in the AG. The majority of the cluster is comprised of right TPJ regions (IPL, SMG, AG and STG; B1 in Fig. 4), with weaker and smaller extensions to the pre- and postcentral gyrus, and the insula and inferior frontal gyrus. On the medial surface, the cingulate cortex (M/PCC) and the precuneus (Prc) also evidenced significant regression values (B2 in Fig. 4), though much smaller than those in S’s data.

Supplementary Fig. S5 (in Supplementary Section 3.1) presents the source images of the one-sample *t-*test between S and the meditators’ group (*n* = 10) regression coefficients. These largely overlap with S’s results, in line with S being a uniquely skilled practitioner. In addition, Supplementary Fig. S5 presents the unmasked (by S’s ROI) group results. These reveal additional occipital and right frontal lateral regions which evidence significant beta band regression effects, interpreted as indicating increased attentional resource allocation for producing the deeper meditative SB states (Saggar *et al.*, 2012; see Supplementary Material, Section 3.1 for a more detailed discussion).

## Discussion

The current study is the first to directly and ecologically tap SSPs, allowing uniquely-trained abilities and phenomenology to guide neuroscientific design and analysis. Together with our previous publication (Ataria *et al.*, 2015), we demonstrate the graded rather than all-or-nothing nature of SSPs on both the experiential and neural levels. Our current results highlight two important findings regarding the neural mechanisms of SSPs. First, we demonstrated that beta oscillations are part of the neural processes associated with changes in the SB. Second, we showed that these modulations can be localized to mainly two anatomical regions in the lateral and medial parietal brain.

The TPJ region was the largest and most pronounced. The TPJ has been shown to play a significant role in self-related paradigms such as self-location (Ionta *et al.*, 2011; Lenggenhager *et al.*, 2011; Blanke, 2012; Guterstam *et al.*, 2015), self-awareness as part of the default mode network (Northoff *et al.*, 2006; Buckner *et al.*, 2008), agency and ownership (Farrer and Frith, 2002; Farrer *et al.*, 2003, 2008; Tsakiris *et al.*, 2010; Dor-Ziderman *et al.*, 2013; Kühn, *et al.*, 2013; Chambon *et al.*, 2014; Khalighinejad and Haggard, 2015), egocentric perspective (Creem *et al.*, 2001; Wraga *et al.*, 2005; Easton *et al*, 2009) and first-person perspective taking (Ruby and Decety, 2001; Vogeley *et al.*, 2004; Ionta *et al.*, 2011). In addition, the literature on out-of-body experiences (OBE), where the unity of self and body is disrupted, and in particular studies where such experiences are produced using full-body illusions (Blanke and Metzinger, 2009), have been linked to the constructs of self-location, agency and ownership, egocentric/first-person perspective, as well as to the TPJ (Blanke *et al.*, 2002; Blanke, 2005a, 2005b; Arzy *et al.*, 2006; Ridder *et al.*, 2007). The right lateralization of the results is aligned with the literature, as lesions leading to OBEs are usually to be found in right parietal regions (Ionta *et al.*, 2011). In addition, studies of multisensory integration of bodily self-awareness (Blanke, 2005b; Arzy *et al.*, 2006; Ionta *et al*, 2014), and the sense of agency (Ruby and Decety, 2001; Farrer *et al.*, 2003; Decety and Lamm, 2007), report effects either more pronounced, or limited to, the right hemisphere.

On the medial surface, the highlighted MPC region is a well-established region mediating self-awareness, reaching back to the original studies of the default mode network (Raichle *et al.*, 2001), and supported by large-scale quantitative meta-analyses of brain imaging studies on self-processing (Northoff *et al.*, 2006; Buckner *et al.*, 2008; Schneider *et al.*, 2008; Kim, 2012). Within the self-network, while the medial prefrontal regions were shown to be involved in self-evaluation (Kwan *et al.*, 2007; Luber *et al.*, 2012), the medial parietal region has been suggested to code for the integration of self-referential stimuli within the context of one’s own person (Lou *et al.*, 2004; Northoff and Bermpohl, 2004), and for the experience of self-identification (Brewer *et al.*, 2013; Garrison *et al.*, 2013; Josipovic, 2013). In addition, the MPC was found to play a major role in studies directly relevant to minimal self-processing, but coming from diverse directions, including fMRI neuroimaging (Araujo *et al.*, 2015), neurophenomenology (Dor-Ziderman *et al.*, 2013), minimally-conscious patients (Laureys *et al.*, 2004), lesion patient studies (Philippi *et al.*, 2012), as well as brain synchrony (Lou *et al.*, 2010).

We interpret these findings as indicative of two concurrent neurofunctional processes which give rise to the experience of diminished SB. The first is freeing conscious awareness from its habitual identification with a self via suppression of the integrative aspect of the self-network in the MPC. The second is a disruption of the self-body unity allowing awareness a measure of flexibility regarding its habitually perceived egocentric location and perspective. Enacting only the former process may result in an OBE type of experience where a spatial shift in the sense of self is induced (Ionta *et al.*, 2011; Blanke, 2012). However, in such cases, the sense of there being a self versus world does not change, only the boundaries are remapped. In other words, the brain maintains its habitual tendency of enacting SSPs and separating the field of experience into self/nonself.

The current setup does not allow inferring causality; however, there is evidence that the MPC can be trained to decrease its activity, as reported in long-term meditators (Brefczynski-Lewis *et al.*, 2007; Brewer *et al.*, 2011; Taylor *et al.*, 2011; Pagnoni, 2012; Berkovich-Ohana *et al.*, 2013; Dor-Ziderman *et al.*, 2013; Marzetti *et al.*, 2014). This suggests that the volitional attenuation of the MPC is a mechanistic target of mindfulness meditation (Brewer and Garrison, 2014). Relying on phenomenological and fMRI data, Josipovic (2013) suggested that the MPC is significantly involved in modulating the fragmentation of experience into subjective versus objective, or self versus other, in meditators. Neurophysiological results tie such states, though not exclusively, to beta band cortical desynchronization (Lehmann *et al.*, 2012; Saggar *et al.*, 2012; Dor-Ziderman *et al.*, 2013; Muthukumaraswamy *et al.*, 2013; Hinterberger *et al.*, 2014; Hauswald *et al.*, 2015).

The current results indicate that the beta band is associated with modulating SSPs. It has been suggested, based on converging evidence from studies of the motor system and related pathophysiology as well as top-down mechanisms involved in cognitive and perceptual processing, that beta band activity (BBA) is related to the maintenance of the current motor/cognitive set. Thus, enhanced BBA signals the intention or prediction of maintaining the status quo while suppression of BBA is argued to signal the opposite—the intention and prediction for disruption of the *status quo* (Engel and Fries, 2010). Applying this hypothesis to the current study, one’s normal, default SSPs gives rise to a natural and powerful subjective state of SB that is rarely perturbed. The SB stems from early constantly adapting evolutionary needs (Llinás, 2001; Damasio, 2010), and as such, involves primitive neural mappings which can be argued to constitute the lowest level of subjective experience and indeed consciousness (Damasio and Carvalho, 2013). Consequently, volitionally manipulating this powerfully implanted SB and producing attenuated and even null states of SB would constitute a gross disruption of the habitual cognitive-experiential *status quo*, and would thus, necessitate a marked reduction in BBA.

The graded nature of SSPs and its trainability leading to the loss of the distinction between “self” and “world” point to a self which is constructed and continuously remade by particular and transient neural processes. That the self is a unitary entity only in the phenomenal sense is an idea which is not alien to the neurocognitive literature. It has been discussed already by William James in the chapter “Consciousness of Self” in his “Principles of Psychology” (James, 1890), and is the topic of numerous current books authored by influential neuroscientists, psychologists, and philosophers (e.g., Llinás, 2001; Metzinger, 2004; Damasio, 2010; Hood, 2012; Harris, 2014). This view of transient selfhood is aligned with the view held by Eastern meditative traditions (see Ganeri, 2012; Thompson, 2015 for an in-depth discussion), and Buddhist ones in particular, which have long claimed that the self is entirely a constructed habit, a mental content, a dominant thought, and not a reality (Dalai Lama, 1997; Ekman *et al.*, 2005; Nydahl, 2008; Austin, 2009). In these traditions, the conceptualization as well as actual experience of reality beyond duality is immensely important (Dunne, 2011; Josipovic, 2013). This understanding remains central in the current widespread theory and practice of mindfulness, a current Buddhist development (Williams and Kabat-Zinn, 2011), and holds a central position in scientific neuropsychological conceptualizations of mindfulness (Lutz *et al.*, 2007; Holzel *et al.*, 2011; Vago and Silbersweig, 2012; Baird *et al.*, 2014; Dahl *et al.*, 2015; Tang *et al.*, 2015). Highly-accomplished mindfulness meditators, through years of long practice, encounter, become familiar with and gain intimate knowledge of states where the self–world separation dissolves, giving rise to a non-dual awareness. Thus, they may develop the abilities to volitionally manipulate such states on demand and even under experimental settings, such as while being scanned by brain imaging devices (Lutz *et al.*, 2004; Josipovic *et al.*, 2011; Dor-Ziderman *et al.*, 2013). This fact reinforces Varela’s (1996) suggestion of using meditators as neurophenomenological subjects, in particular regarding themes concerning the self.

The distinct neural characterization and plasticity of SSPs may hold practical value for clinical populations suffering from abnormal SB. For example, during trauma, an involuntary shift from the regular daily experience to a sense of rigid and closed SB may be enacted as a defense mechanism (Ataria, 2013, 2014). In addition, depersonalization disorder (DPD) is characterized by a sense of unreality about the self and the world, and thus directly reflects a disturbed mode of SSP (Sierra and David, 2011). Gray matter changes in the frontal, temporal, and parietal lobes (both lateral and medial) are associated with DPD (Sierra *et al.*, 2014), as well as hyperactivation of the TPJ (Simeon *et al.*, 2000). Finally, preliminary studies suggest that noninvasive stimulation of the right TPJ may be a therapeutic option for DPD. Repetitive transcranial magnetic stimulation over the course of weeks in DPD patients resulted in an impressive reduction of symptoms in about half of the patients (Mantovani *et al.*, 2011). The present study’s novel contribution in highlighting the associated frequency band and electrodes' spatial location may further aid clinicians both in early detection of SSPs’ abnormalities, as well as the design of neurofeedback interventions (Bagdasaryan and Quyen, 2013), in particular, interventions utilizing immersive virtual reality environments which allow ecologically relevant learning (Gruzelier *et al.*, 2010).

The current study suffers from a number of drawbacks. The first is the study’s design which lacked randomization in the ordering of conditions. Attempts were made to control for this flaw (see section “Methods,” “Sensor space analysis,” and “Analysis and statistics”; and Supplementary Material Sections 2.1 and 2.2), which was largely due to the neurophenomenological nature of the study which imposed certain restrictions both in terms of facilitating the production of these rare states, as well as the requirements of phenomenological data collection. As the concepts and processes engaged with are difficult to operationalize, and similar studies are scarce, refining methodology is a task for future experimentation. A further drawback is that the prior neural hypothesis is based on a single subject. While this drawback was partly overcome by corroborating the results in a larger group, the degree to which the results can be generalized to wider, non-meditative populations is uncertain. As such, the study is preliminary. In addition, the study’s setup does not support inferring causality (that the modulation of beta oscillations causes changes in the SB). We cannot completely rule out the possibility that the reported changes may be due to downstream effects of other processes, or could be purely epiphenomenal. Nevertheless, despite these limitations, this proof-of-concept study illustrates that studying subtle but profound aspects of self-identity is tenable by incorporating first-person data into neuroimaging experimental protocols. We hope it will spark further robust examination of the brain mechanisms, trainability, and clinical applicability of SSPs.

## Supplementary Data

Supplementary data is available here.

## Supplementary Material

Supplementary Data
