# Supplementary material for: Self-specific processing in the meditating brain: a MEG neurophenomenology study
Source: Neurosci Conscious. 2016 Oct 10;2016(1):niw019. doi: 10.1093/nc/niw019 (PMC6210398; doi:10.1093/nc/niw019)
Supplement: Supplementary Data [file niw019_supplementary_data.zip › SuppMaterial.docx]

**SUPPLEMANTARY MATERIAL**

1. **INTRODUCTION**
   1. **Linking the nine SB phenomenal categories to self-awareness**

The relation between S’s nine phenomenological categories and the top-down literature-based self-mode categories requires some clarification. To begin, it needs to be acknowledged that there is a certain overlap between the category of ‘self’ and some of the other categories, in particular those of ‘bodily experience’, ‘location’, ‘agency’, and ‘ownership’ – which are often regarded as dimensions of self-experience. However, as discussed in our already published work on S’s phenomenology (Ataria, Dor-Ziderman, & Berkovich-Ohana, 2015), the process of phenomenal category formation was very much reliant on the language and descriptions provided by S. Staying as close as possible to the descriptions provided by S, who is very precise in his descriptions, the categories ‘formed themselves’ in a bottom-up manner (as dictated by the grounded theory approach) and took their present form (see Table 1). As Ataria et al. (2015) and the present paper are two aspects of one project – aiming to bridge phenomenal and neural aspects of the sense of boundaries, the same nine phenomenological categories are maintained.

Table 1 (in the main text) presents the results of the interview, demonstrating how the nine different categories altered (from strong to non-existent) during the three stages of meditation. As table 1 indicates, while all the categories underwent change in the various states of SB, their existence and strength at each stage differs. This observation led to a higher level of organization, grouping the categories into the following threefold structure depicted in Figure S1 below.

1. Default state: The first stage is characterized by a relatively strong sense of time, location, self, agency (control), and ownership. Thus, it is possible to suggest that a “normal” SB is accompanied by these, and a “normal” state of consciousness (as opposed to altered state of consciousness) maintains the structure presented below in Supplementary Figure 2.
2. Dissolving: When the SB dissolves, several categories weaken significantly, including the sense of time, location, self, agency, and ownership (see table 1). Yet other categories remain the same, including the sense of being at the center (with one's body as a reference point), and mineness. Thus it appears that when the SB starts to dissolve, the sense of being within one’s body as a center of reference remains, along with a weak sense of ownership; even though the structure becomes less steady, it does not collapse altogether.
3. Disappearing: When the SB disappears (and the level of flexibility reaches its height), on the one hand further categories of the experience completely vanish, including the senses of: internal versus external, time, location, self, agency, ownership, and center. On the other hand, some general bodily feelings (proprioception, kinesthesia etc.) remain, in addition to the touching-touched structure. Hence, even when the SB drops to zero, we retain some bodily feelings along with a primitive – not object- but rather world-oriented – touching-touched structure. Thus, it seems reasonable to suggest that boundaries, in contrast to the sense of boundaries, can be described in terms of general bodily feelings. These bodily feelings represent, in turn, the internal side of the equation and the primitive engagement (touching/touched) with the world (not object oriented) that represents the external side of the equation.

Figure S1: Hierarchical structure of the three different levels of SB

This model depicts a hierarchical structure of the three different levels of SB – it demonstrates a higher level of abstraction of the data. Red represents the SB1 (Default state); green represents SB2 (Dissolving); and purple represents SB3 (Disappearing). The more flexible the SB becomes, the more the dimensions of experience dissolve until they finally disappear. Yet several dimensions do not disappear altogether on the 3^rd^ level. These represent actual bodily borders, as opposed to the SB.

The threefold structure of the SB experience is useful in relating the present results to other models of consciousness and in particular to neurobiological accounts of consciousness, thus allowing operationalization of these parameters using existing conceptual formulations. The model presented here is compared to Damasio’s threefold neurobiological account of the sense of self (A. R. Damasio, 1999; A. Damasio, 2010), one of the most detailed and comprehensive account currently available (it is also compatible with other influential conceptual frameworks such as that of Gallagher, 2000). According to Damasio’s account, self-awareness is a pre-requisite for consciousness and can be divided into 3 levels. The first is the *proto-self*, a preconscious biological phenomenon concerning the bodily state of the organism (including the internal milieu, viscera, vestibular system, and musculoskeletal frame). This level unconsciously maps, moment by moment, the physical state of the organism and creates a first-order representation of current body states. The description of the third, disappearing level, on which only the touching-touched structure and bodily feelings remain, may reflect a conscious experience of some normally hidden proto-self processes. These actual bodily borders, knowing “what is inside and what is outside” of the body, are core biological processes necessary for survival and normal functioning and are thus maintained even in the disappearing stage.

Damasio’s second level is that of the *core-self*, a conscious and knowing self, based on the feeling state arising when the organism’s proto-self interacts with first order sensory maps that represent objects. This interaction creates changing second order mental maps. Hence the core-self is a transient, conscious state, continuously generated through encounters with objects and immersed in experience. On this level, subjectivity develops for the first time, there is a (minimal sense of) “I” in relation to a “world”. In our threefold model, this level may be paralleled to the stage at which the SB dissolves, a stage which is strongly embodied with a weak sense of ownership and agency. Finally, Damasio’s third level is the extended/narrative-self, which is created from the core-self and extends in time and space. It is dependent on extended-consciousness, which is involved in the formation of enduring experiential memories, attention, language, and location, and its inevitable concomitant is the personal identity. This level is parallel to the default SB level, at which agency, ownership, sense of self, time, and location function in an ordinary manner and are woven into the very fabric of the physical and social world.

1. **SENSOR LEVEL**
   1. **Controls for stage 1 sensor-level analysis**
      1. *Re-grouping SB conditions by order of appearance*

In order to partially control for the fixed order of SB production, the data was re-grouped into conditions based on temporal order alone, and an identical regression analysis was performed on the newly-grouped data. As can be seen below, in the regrouped setup the significant beta-band results disappear. This indicates that the regression results cannot be explained by presentation order alone (the fact that in every block, SB1 precedes SB2, which precedes SB3).

Figure S2: Re-grouping the conditions by order of presentation


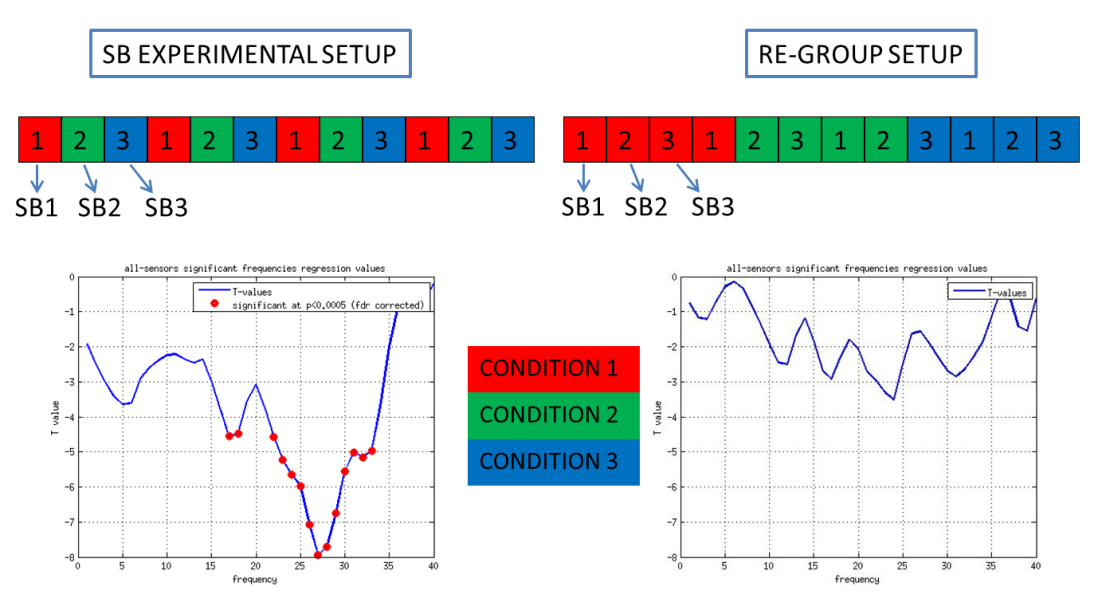
 Controlling for fixed order of conditions. Plots of regression values for each frequency (1-40 Hz) grouped either by SB state (left) or by order of presentation regardless of SB state (right). Colors indicate grouping into conditions for regression analysis, of either the original data (left – SB1 (red), SB2 (green), and SB3 (blue)) or the regrouped data (right – first third (red), second third (green), last third (blue)).

- - 1. *Baselining the data*

The one minute rest periods in each block were used for baseline correction of the SB data. This further analysis was performed in order to ensure that (1) frequencies changes in spectral data power were not masked by power changes in much lower frequencies; and (2) that the reported power changes did not reflect a return to equilibrium rather than genuine increases/decreases in power. We computed the absolute power for each frequency (1-40 Hz) for each 1 minute rest epoch (total of 4), and then subtracted these values from each SB state in the corresponding block. Supplementary Figure 3 below shows the FFT coefficients themselves across frequencies before (above) and after baseline correction (below). As can be seen, the reported results do not change in a meaningful way (comparable regression results – see upper right top and bottom), in particular within the relevant high beta-band frequency (22-33 Hz, see enlarged sections of power spectrum). Both figures reveal alpha frequency band significant differences between SB states. However, these are not relevant to the phenomenologically-guided regression results (that SB1 > SB2 > SB3) presented in the main body of text.

Figure S3: Spectral power before and after baseline correction


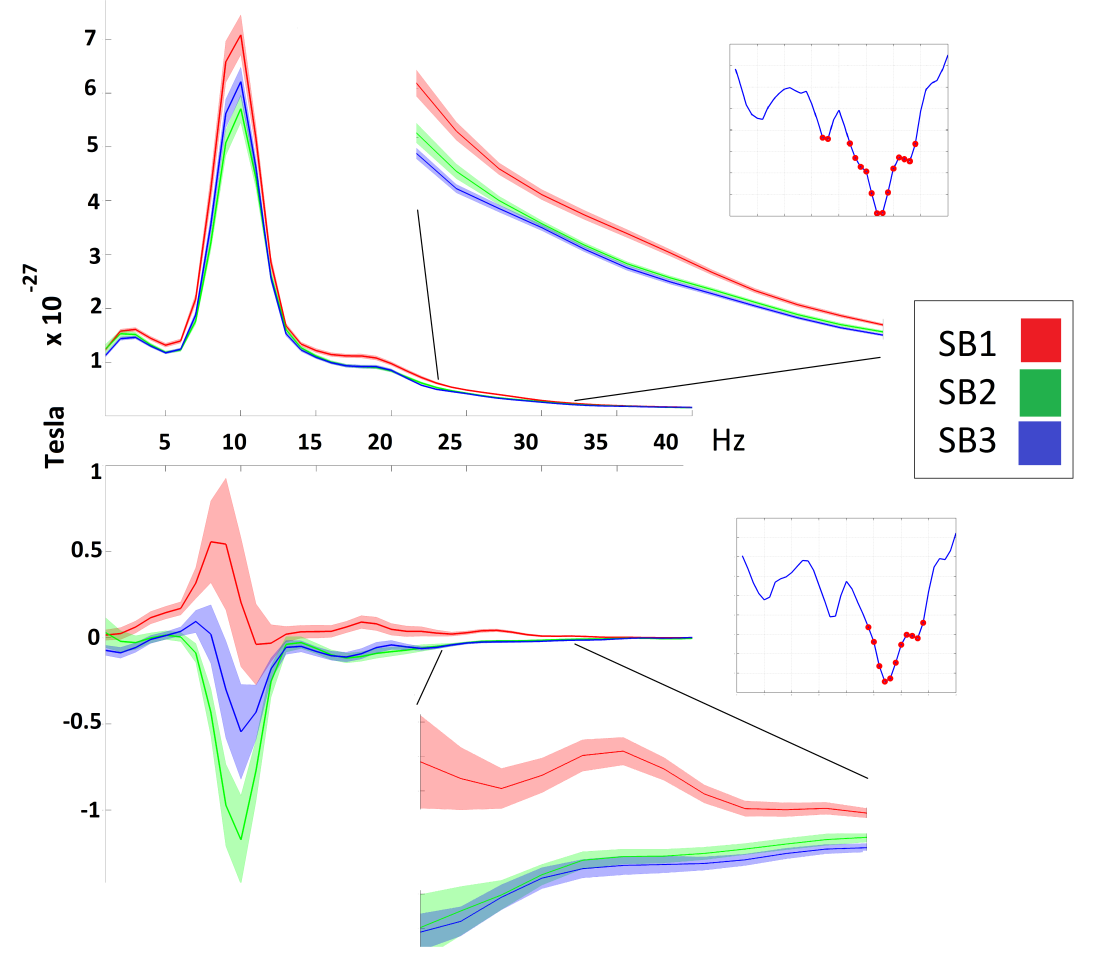
 Spectral distribution before (above) and after (below) baselining the data by each blocks’s 1-min rest period. Solid lines indicate mean power and shaded areas plus/minus standard errors. Frequencies of interest (22-33 Hz) are shown in an enlarged view. Statistical figures of regression results (averaged over all sensors) appear in upper-right corners, above (before baselining) and below (after baselining).

- 1. **Control blocks for stage 2 sensor-level analysis**
     1. *Time and Space control analyses*

To address the lack of randomization in stage 2, we analyzed two other similarly ordered and designed tasks (see Supplementary Figure 4 below) regarding the the *sense of time* (Try to be in the present moment/past/out of time) and *sense of space* (Try to be here/elsewhere/not in the center of space), carried out by this same group of subjects in a different part of the same experiment (reported in Berkovich-Ohana, Dor-Ziderman, Glicksohn, & Goldstein, 2013). If the reported beta band effects were indeed caused by the fixed order of blocks, we would expect to get a similar beta band effect in these tasks as well. We conducted a whole-head analysis (described in the section 2.2.2). No beta band effects were found for either *time* or *space*, suggesting that the reported beta band effects could not be attributed to design structure (section 3.2.1).

Figure S4: Setup of time and space control blocks


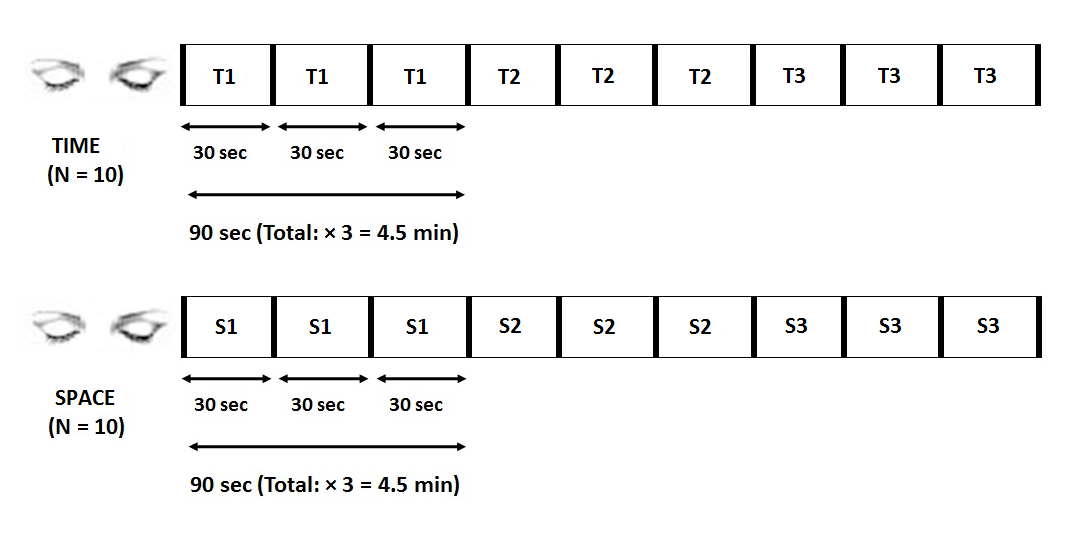
 Timing and sequence of conditions in the MEG sessions of *time* and *space*. T1-3: “Then”/”Now” /”Timeless”; S1-3:“There”/”Here”/”Spaceless”. All epochs were initiated by an auditory cue.

1. **SOURCE LEVEL**
   1. **Additional comparison of source images**

Figure S5 below presents source images of different statistical comparisons (rows) and brain views (columns). The first and third row present data already shown in the main text (S’s and the masked group’s images, Figures 3 and 4 respectively); the second row a 1-sample *t-test* between S and the meditators group (n=10) regression coefficients; and the 4^th^ row the unmasked (by S’s ROI) group results.

Figure S5: Source images of different comparisons


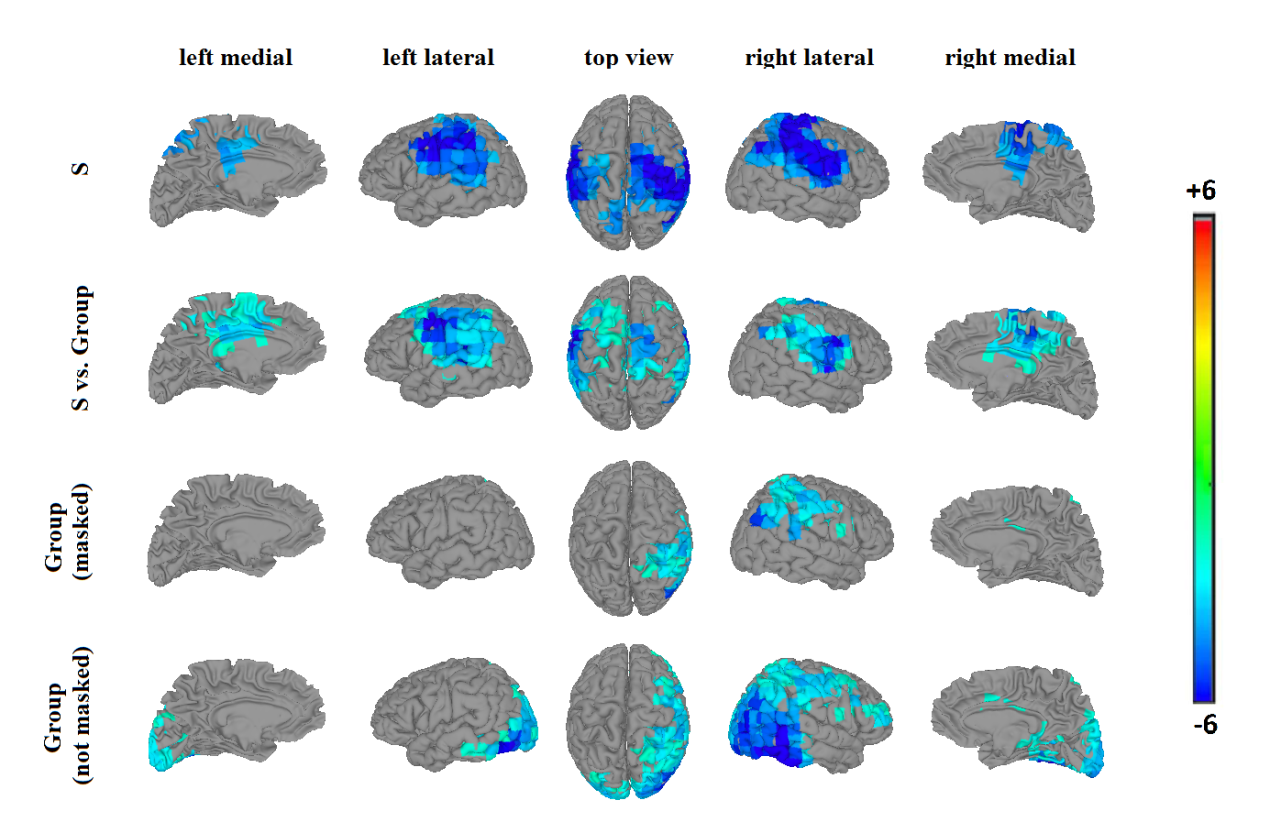


Source images of different statistical comparisons overlaid on SUMA 3-D cortical surface model. From top to bottom (y-axis): S’s (n=1) regression statistical image (as in Figure 3), 1-sample *t-test* between S and the meditators group (n=10) regression coefficients; the group’s (n=10) regression statistical image (as in Figure 4) masked by S’s ROI; unmasked group’s regression image. From left to right (x-axis): Different brain views. All the images are significant at *p*<0.0005 (Monte Carlo corrected). Color bar indicates *t* value degree.

The comparison between S and the group (2^nd^ row) can be interpreted as confirming the expertise of S in relation to the group. The significant regions found for S largely overlap with the regions found when comparing his image to the group, indicating more significant decreases in beta activity relative to the group. However, these interpretations need to be considered with caution, due to differences in SNR level and task design of stage 1 and 2.

The unmasked group data (bottom row) reveals additional regions that show significant beta band regression values (narrative>minimal>selfless) to those already shown in the group data masked by S’s ROI (Figure 4 in the main text, and 3^rd^ row images in present figures). These further regions are mainly occipital, but also right fronto-lateral (compare top and bottom right images). Reductions in meditative state-related beta-band power over posterior and anterior scalp regions have been observed in the literature on experienced meditators (Hinterberger, Schmidt, Kamei, & Walach, 2014; Saggar et al., 2012), and have been tied to the conscious experience of ‘emptiness’ (former study) and to extent of meditation practice (both studies). Interpreting these results is somewhat problematic due to the ‘thin’ phenomenology gathered in stage 2. However, These widespread decreases in beta activity in areas attributed to motor-tasks, vision and cognition have been interpreted elsewhere as reflecting increased cortical activation of sensory-related attentional networks (Saggar et al., 2012). That these regions do not come up in S’s data may be due to his very high level of expertise and familiarity in producing these deeper SB states, thus not requiring allocation of additional attentional resources to sustain attention as the SB dissolves (Brefczynski-Lewis, Lutz, Schaefer, Levinson, & Davidson, 2007).

**REFERENCES**

Ataria, Y., Dor-Ziderman, Y., & Berkovich-Ohana, A. (2015). How Does it Feel to Lack a Sense of Boundaries? A Case study of a long-term Mindfulness Meditator. *Consciousness and Cognition*.

Berkovich-Ohana, A., Dor-Ziderman, Y., Glicksohn, J., & Goldstein, A. (2013). Alterations in the sense of time, space, and body in the mindfulness-trained brain: a neurophenomenologically-guided MEG study. *Frontiers in Psychology*, *4*, 912. doi:10.3389/fpsyg.2013.00912

Brefczynski-Lewis, J. A., Lutz, A., Schaefer, H. S., Levinson, D. B., & Davidson, R. J. (2007). Neural correlates of attentional expertise in long-term meditation practitioners. *Proceedings of the National Academy of Sciences of the United States of America*, *104*, 11483–11488. doi:10.1073/pnas.0606552104

Damasio, A. (2010). *Self Comes To Mind: Constructing the Conscious Brain*. New York: Pantheon.

Damasio, A. R. (1999). *The feeling of what happens: Body and emotion in the making of consciousness*. New York: Harcourt.

Gallagher, S. (2000). Philosophical conceptions of the self: implications for cognitive science. *Trends in Cognitive Sciences*, *4*(1), 14–21. doi:10.1016/S1364-6613(99)01417-5

Hinterberger, T., Schmidt, S., Kamei, T., & Walach, H. (2014). Decreased electrophysiological activity represents the conscious state of emptiness in meditation. *Frontiers in Psychology*, *5*, 99. doi:10.3389/fpsyg.2014.00099

Saggar, M., King, B. G., Zanesco, A. P., Maclean, K. a, Aichele, S. R., Jacobs, T. L., … Saron, C. D. (2012). Intensive training induces longitudinal changes in meditation state-related EEG oscillatory activity. *Frontiers in Human Neuroscience*, *6*(September), 256. doi:10.3389/fnhum.2012.00256
